# TFEB activation hallmarks antigenic experience of B lymphocytes and directs germinal center fate decisions

**DOI:** 10.1038/s41467-024-51166-3

**Published:** 2024-08-14

**Authors:** Matthias Münchhalfen, Richard Görg, Michael Haberl, Jens Löber, Jakob Willenbrink, Laura Schwarzt, Charlotte Höltermann, Christian Ickes, Leonard Hammermann, Jan Kus, Björn Chapuy, Andrea Ballabio, Sybille D. Reichardt, Alexander Flügel, Niklas Engels, Jürgen Wienands

**Affiliations:** 1 Institute of Cellular & Molecular Immunology, University Medical Center Göttingen, Göttingen, Germany; 2 Institute for Neuroimmunology and Multiple Sclerosis Research, University Medical Center Göttingen, Göttingen, Germany; 3 Department of Medical Hematology and Oncology, University Medical Center Göttingen, Göttingen, Germany; 4 Department of Hematology, Oncology, and Tumor Immunology, Charité, Campus Benjamin Franklin, University Medical Center Berlin, Berlin, Germany; 5 Institute of Cardiovascular Physiology, University Medical Center Göttingen, Georg August University, Göttingen, Germany; 6 Telethon Institute of Genetics and Medicine (TIGEM), Naples, Italy; 7 Department of Translational Medical Sciences, Federico II University, Naples, Italy; 8 Department of Molecular and Human Genetics, Baylor College of Medicine, Houston, USA; 9 Jan and Dan Duncan Neurological Research Institute, Texas Children’s Hospital, Houston, USA

**Keywords:** B cells, B-cell receptor, Signal transduction, Gene regulation in immune cells, Immune cell death

## Abstract

Ligation of the B cell antigen receptor (BCR) initiates humoral immunity. However, BCR signaling without appropriate co-stimulation commits B cells to death rather than to differentiation into immune effector cells. How BCR activation depletes potentially autoreactive B cells while simultaneously primes for receiving rescue and differentiation signals from cognate T lymphocytes remains unknown. Here, we use a mass spectrometry-based proteomic approach to identify cytosolic/nuclear shuttling elements and uncover transcription factor EB (TFEB) as a central BCR-controlled rheostat that drives activation-induced apoptosis, and concurrently promotes the reception of co-stimulatory rescue signals by supporting B cell migration and antigen presentation. CD40 co-stimulation prevents TFEB-driven cell death, while enhancing and prolonging TFEB’s nuclear residency, which hallmarks antigenic experience also of memory B cells. In mice, TFEB shapes the transcriptional landscape of germinal center B cells. Within the germinal center, TFEB facilitates the dark zone entry of light-zone-residing centrocytes through regulation of chemokine receptors and, by balancing the expression of Bcl-2/BH3-only family members, integrates antigen-induced apoptosis with T cell-provided CD40 survival signals. Thus, TFEB reprograms antigen-primed germinal center B cells for cell fate decisions.

## Abstract (web summary)

B cell receptor activation leads to contrary outcomes in the absence and presence of co-stimulation. Here, the authors show transcription factor EB acts as a B cell receptor-controlled rheostat that balances activation-induced cell death with co-stimulatory rescue signals, collectively reprograming antigen-primed germinal center B cells for fate decisions.

## Introduction

The initiation of humoral immune responses by B lymphocytes requires two consecutive activation steps to drive their terminal differentiation into antibody-secreting plasma cells or long-lived memory B cells^1,2^. First, ligation of the B cell antigen receptor (BCR) triggers a series of intracellular signaling events that become functionally complemented by co-stimulatory signals delivered through co-receptors, most notably CD40^3,4^. In fact, B cell priming without secondary co-stimulation can result in cell silencing or even deletion (‘death-by-default’), reflecting the existence of tolerance mechanisms to prevent the production of auto-antibodies^5^. The opposing roles of BCR ligation in promoting versus suppressing B cell activities remain mechanistically elusive even though the BCR-proximal signaling machinery including protein kinases and adapter elements has been deciphered to quite some extent^6,7^. A diverse set of BCR-distal events governs the reorganization of B-lymphoid gene expression profiles, culminating into biological responses, such as expression of co-stimulatory surface receptors, up-regulation of MHC class II molecules or the induction of migration and homing of B cells to germinal centers (GC) in lymphatic tissues^8^. Key to genetic reprogramming is the inducible translocation of transcriptional regulators from the cytosol into the nucleus. Prominent shuttling elements are the transcription factor families nuclear factor of κ-binding (NF-κB) and nuclear factor of activated T cells (NFAT)^9^. Indirect regulation of transcription factor activity is exemplified by the nuclear translocation of cytosolic serine/threonine kinases e.g., MAPKs like ERK, which phosphorylate and thereby activate transcription factors of the Jun/Fos, Elk or Ets families, among others^10^.

While initial T cell:B cell interactions take place at the boundary of follicles and the T cell zone, T follicular helper (T_FH_) cells within GCs of secondary lymphoid follicles provide the main source of secondary B cell co-stimulation^11,12^. To physically interact with and receive second signals from T_FH_ cells, B cells internalize and process BCR/antigen complexes, and present resulting peptide fragments on the cell surface in the context of MHC class II molecules, whose expression increases upon B cell priming. This results in the formation of the ‘immune synapse’ and the engagement of the most prominent B cell co-activator CD40^12^. To receive co-stimulatory signals, antigen-primed B cells are guided by cytokines, which control their migration and GC entry^13^. During the GC reaction, antigen-primed B cells compete for T_FH_-provided secondary signals, the magnitude of which directs further B cell differentiation fates. The recall of class-switched memory B cells is less dependent on additional signal input^14^. However, sole BCR engagement without a second ‘go signal’ in a time-limited window progressively deprives primed B cells of metabolic supply through mitochondria and glycolysis, leading to cell death rather than proliferation or differentiation^15,16^.

The sequential two-step stimulation requirement is part of a mechanistically mysterious signal integration network, underlying the establishment and maintenance of immune tolerance^17^. How BCR signaling prepares the B cell for death-by-default on the one hand and provides a possible exit strategy on the other is not known, but likely involves transcriptional re-programming^15,16,18,19^. Here, we present an unbiased and comprehensive inventory of B-lymphoid nuclear logistics, unveiling transcription factor EB (TFEB), a member of the microphthalmia-inducing transcription factor (MiTF) family^20,21^, as a rheostat that destines BCR-stimulated B cells to apoptosis and simultaneously triggers the expression of a tool kit for receiving secondary rescue signals.

## Results

### Kinetic profiling of the ‘nuclear translocatome’ in resting and BCR-activated B cells

**Fig. 1:**
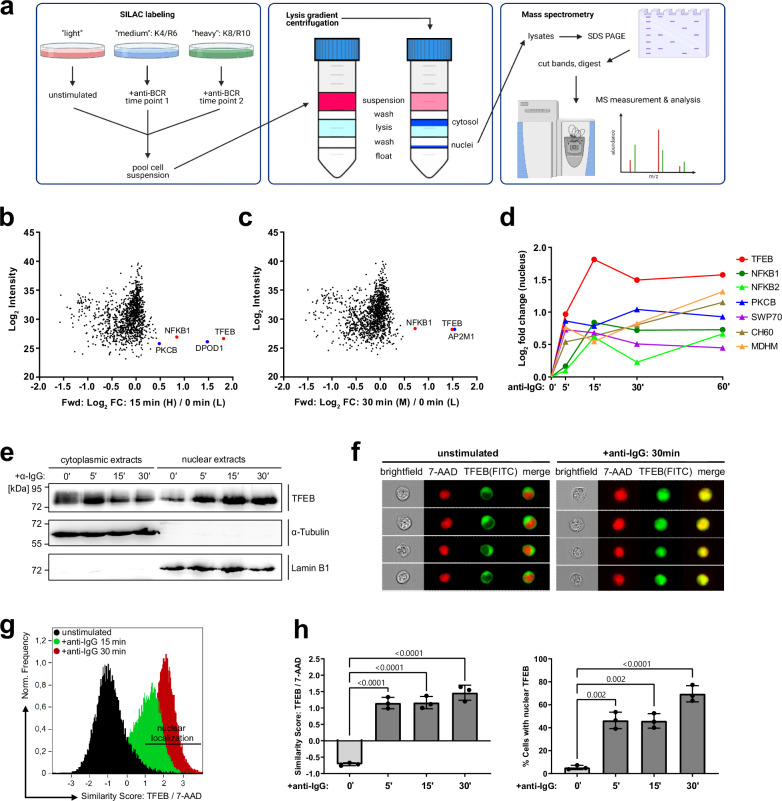
Proteomic profiling of B-lymphoid nuclear logistics reveals TFEB as a BCR-inducible element. **a** Schematic representation of the ‘translocatome’ approach. IIA1.6 B cells were metabolically labeled via SILAC using three distinct combinations of isotope-marked lysine (K) and arginine (R), and either left untreated or BCR-stimulated for multiple time points (left). Pooled cells were fractionated by iodixanol-based lysis gradient centrifugation (middle). Nuclei were lysed and nuclear proteins were quantified by LC/LC tandem mass spectrometry (right). This graphical overview was created with BioRender.com under a Creative Commons Attribution-NonCommercial-NoDerivs 4.0 international license. **a**–**c** Triple SILAC MS analysis of BCR-induced nuclear translocation. **b**, **c** Scatter plots representing log_2_-fold enriched nuclear proteins following 15 and 30 min of BCR stimulation plotted against the log_2_ signal intensity. Proteins significantly enriched at two-time points are highlighted in blue, and significantly enriched proteins at ≥ 3-time points are marked in red. **d** Nuclear translocation kinetics of significantly enriched proteins detected at all indicated time points of BCR stimulation. Circles and triangles indicate proteins identified under ‘forward’ or ‘reverse’ SILAC labeling conditions. **e** IIA1.6 B cells were left untreated or BCR-stimulated for the indicated time periods, fractionated via iodixanol-based lysis gradient centrifugation and the subcellular distribution of TFEB was analyzed by immunoblotting with anti-TFEB antibodies. Successful subcellular fractionation was confirmed by immunoblotting with antibodies against tubulin and lamin B1 as cytosolic and nuclear envelope marker proteins, respectively. Relative molecular masses of marker proteins are indicated on the left in kDa. **f**–**h** Resting or BCR-stimulated IIA1.6 B cells were fixed and stained with rabbit anti-TFEB and anti-rabbit-FITC antibodies. Nuclei were counterstained with 7-AAD. Nuclear translocation kinetics of TFEB was assessed by imaging flow cytometric analysis of 2 × 10^4^ cells and is shown in (**f**) as representative images depicting untreated versus BCR-stimulated cells, in (**g**) as histograms depicting the similarity co-localization scores of TFEB (FITC) versus 7-AAD in (**h**) as mean similarity score of TFEB/7-AAD and as defined by the percentage of cells with similarity score of TFEB/7-AAD ≥ 1. Data is depicted as mean ± SD of *n* = 3 independent experiments. Statistical significances were calculated using one-way ANOVA and corrected for multiple testing via Tukey’s method. Source data are provided as a Source Data file.

In search for yet unexplored transcriptional regulators of primed B cells, we applied a subcellular fractionation protocol^22^, allowing for the cross-contamination-free isolation of cytosolic and nuclear proteins that were then identified and quantified by SILAC-based tandem mass spectrometry, revealing the B-lymphoid ‘translocatome’. Briefly, murine IIA1.6 B cells expressing an IgG-BCR were metabolically labeled with three distinct combinations of isotope-marked amino acids. The individual cell batches were left untreated or BCR-stimulated for various time points. Batches were pooled and subjected to lysis gradient centrifugation, resulting in cytosolic and nuclear protein fractions that were finally identified by mass spectrometry (Fig. 1a). A possible influence on the fractions’ purity or composition by the metabolic labeling procedure was excluded through reversely switching the amino acid culture conditions and by an independent replicate (Supplementary Fig. 1a–e). Our approach confirmed the nuclear shuttling of NF-κB family members^9^, protein kinase C isoforms (PKCβ)^23^ and SWAP70, a guanine nucleotide exchange factor^24^. Intriguingly, our ‘translocatome’ analysis uncovered TFEB as a BCR-regulated nuclear resident (Fig. 1b–d, Supplementary Fig. 1a–e). Moreover, when extending our stimulation protocol from 5 to 60 min followed by plotting of the nuclear-shuttling kinetics, TFEB appeared to be the most efficient and dominating translocator compared to all other BCR effectors including NF-κB1 (Fig. 1d). Immunoblot analyses confirmed the rapid and robust nuclear entry of TFEB in response to BCR ligation as well as the absence of cross-contamination among the subcellular protein fractions (Fig. 1e). Furthermore, we visualized the subcellular distribution of TFEB and its dynamic re-organization by multicolor imaging flow cytometry (Fig. 1f–h, Supplementary Fig. 1f). In resting IIA1.6 B cells, TFEB was homogeneously distributed in the cytosol and largely excluded from the nucleus (Fig. 1f). BCR ligation effectively induced TFEB nuclear translocation but residual amounts of cytosolic TFEB were still detectable. The imaging of individual cells was complemented by flow cytometric histogram analysis of the translocation kinetics and the statistical calculation of subcellular similarity scores, representing the spatial signal overlap of cytosolic and nuclear TFEB (Fig. 1g, h). These data revealed that the vast majority of IIA1.6 B cells show inducible TFEB translocation.

From an analytical perspective, our approach of collecting subcellular fractions from differently stimulated cells in combination with the quantitative elucidation of their proteomic profiles turned out to be a powerful method to study the quality and kinetics of nuclear logistics.

### Nuclear translocation of TFEB hallmarks B cell activation and antigenic experience

The multi-domain protein TFEB is best known for its role in lysosomal biogenesis and as a master regulator of autophagy in a number of cell types^25,26^. Little is known about TFEB in B cells, which express much higher amounts of TFEB than any other cell type^27^. We thus corroborated our finding of TFEB being a BCR-regulated nuclear effector by in-depth analysis of various B cell lines as well as primary B cells of murine and human origin. Imaging flow cytometry of murine WEHI-231 and human Ramos B cells, both expressing an IgM-BCR, phenocopied the TFEB response pattern observed for IgG-positive IIA1.6 cells, namely almost absent TFEB in the nucleus of resting cells and near-to-complete nuclear positivity following BCR ligation (Supplementary Figs. 1g–j).

**Fig. 2:**
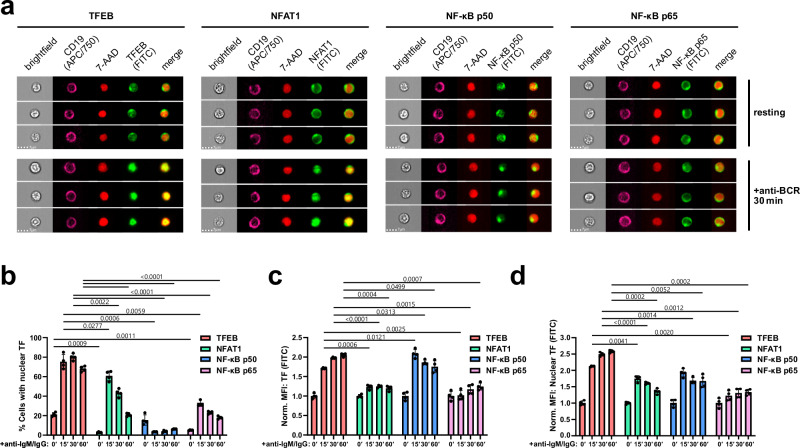
Translocation of TFEB hallmarks B cell receptor signaling. Splenocytes of wild-type C57BL/6 mice were left untreated or stimulated with anti-IgM and anti-IgG for the indicated durations. **a**, **b** TFEB, as well as the classical transcription factors NFAT1, NF-κB p50 and NF-κB p65 was assessed for BCR-induced nuclear translocation by staining the transcription factors together with the nuclear marker 7-AAD as described above. Cells with similarity scores ≥ 1 were defined as cells with nuclear localization of the respective transcription factor. Representative images (**a**) and statistical analysis (**b**) of nuclear translocation. **c**, **d** Expression levels, as defined by the fluorescence intensity of the respective transcription factors, (**c**) within the whole cell and (**d**) within the nucleus. All data are depicted as mean ± SD derived from *n* = 4 biological replicates. Statistical significances were calculated using RM two-way ANOVA and corrected for multiple testing via Dunnett’s method. Source data are provided as a Source Data file.

TFEB activation could also be observed in CD19-positive murine splenic B cells, which showed inducible nuclear translocation (Fig. 2a, b), augmented overall expression (Fig. 2c), as well as increased nuclear abundance of TFEB (Fig. 2d) in response to BCR ligation. In mouse splenic B cells, nuclear deposition of TFEB constituted an even more reliable hallmark of BCR ligation compared to the well-known translocating transcription factors NFAT1, NF-κB p50 and NF-κB p65 (Fig. 2). Moreover, while TFEB was observed to be translocated upon BCR stimulation in every examined cell line, NF-κB p65, ERK, AKT and JNK displayed a marked mobilization in not more than some B cell lines (Supplementary Fig. 2a, b).

**Fig. 3:**
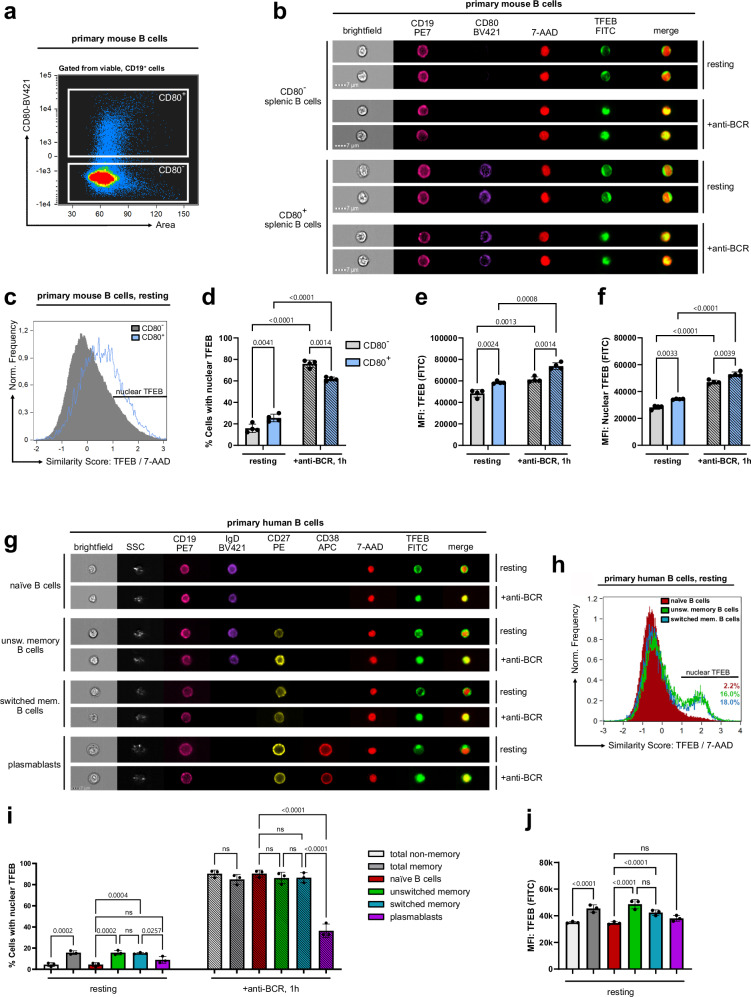
Nuclear TFEB reflects antigenic B cell experience. **a**–**f** Splenocytes of aged-matched wild-type C57BL/6 mice were left untreated or stimulated with anti-IgM and anti-IgG for 60 min. Among splenic CD19^+^ B cells, CD80^-^ B cells and CD80^+^ memory B cells were differentiated through surface staining (**a**). TFEB nuclear translocation was analyzed by imaging flow cytometry as described in Fig. 1. **b** Representative multi-channel images of individual cells. **c** Histograms depicting TFEB/7-AAD similarity scores of CD80^-^ and CD80^+^ B cells in the absence of BCR ligation. **d** Percentages of cells with similarity scores of TFEB/7-AAD ≥ 1 in resting and BCR-stimulated subsets. **e**, **f** Expression of TFEB (**e**) within the whole cell and (**f**) within the nucleus. **g**–**j** Primary human B cells were isolated from the blood of healthy donors and left untreated or BCR-stimulated for 60 min. Localization of TFEB in individual primary human B cell subpopulations is defined by the following cell surface staining patterns. CD19^+^CD27^-^IgD^+^ (naïve), CD19^+^CD27^-^ (total non-memory), CD19^+^CD27^+^ (total-memory), CD19^+^CD27^+^IgD^+^ (unswitched memory), CD19^+^CD27^+^IgD^-^ (switched memory), or CD19^+^CD27^+^IgD^-^CD38^+^ (plasmablasts). The corresponding gating strategy is shown in Supplementary Fig. 2f. **g** Representative multi-channel images of individual cells. **h** Histogram analysis of naïve B cells, or unswitched and switched memory B cells in the absence of BCR ligation. of TFEB (FITC) versus 7-AAD similarity score. **i** Percentages of cells with similarity scores of TFEB/7-AAD ≥ 1 in resting and BCR-stimulated B cell subsets. **j** TFEB expression among resting B cell subsets. All data are depicted as mean ± SD of *n* = 3 independent experiments. Statistical significances were calculated using one-way ANOVA and corrected for multiple testing via Tukey’s method. Source data are provided as a Source Data file.

Next, we assessed TFEB localization and expression in naïve and antigen-experienced murine B cells by means of CD80 co-staining (Fig. 3a–f). CD80 constitutes a well-established marker of B cell activation and memory subsets in both humans and mice^28,29^. In both the CD80-positive and -negative subpopulations, BCR ligation triggered nuclear TFEB translocation (Fig. 3b, d) and increased its overall expression as well as its nuclear abundance (Fig. 3e, f). Even in the absence of ex vivo stimulation, antigen-experienced CD80^+^ B cells exhibited significantly higher TFEB nuclear residency than CD80^−^ cells (Fig. 3c, d). Moreover, TFEB expression (Fig. 3e) and nuclear quantity (Fig. 3f) were elevated in CD80^+^ cells both before and after (re-)stimulation of the BCR. Hence, nuclear TFEB does not only constitute a hallmark of recent BCR stimulation, but also of antigenic experience.

Consistent with our observations in primary mouse B cells and various B cell lines, CD19-positive peripheral blood human B cells showed BCR-induced TFEB localization (Supplementary Fig. 2c, d) as well as elevated TFEB expression levels (Supplementary Fig. 2e). Hence, inducible TFEB nuclear translocation and enhanced expression appear to be general mechanisms of B cell priming. Next, we gated on individual primary human B cell subpopulations (Supplementary Fig. 2f) and monitored their TFEB translocation dynamics (Fig. 3g–j). Spatial similarity analysis showed that, while BCR ligation triggered nuclear translocation of TFEB in all subsets (Fig. 3i), stimulation-independent nuclear positivity was found for both resting unswitched (CD19^+^, IgD^+^, CD27^+^, CD38^−^) as well as switched (CD19^+^, IgD^−^, CD27^+^, CD38^−^) memory B cell pools with similar proportions of around 15%, yet not in naïve B cells (CD19^+^, IgD^+^, CD27^−^, CD38^−^) and plasmablasts (CD19^+^, IgD^−^, CD27^+^, CD38^+^) (Fig. 3h, i). As observed in B cells of murine origin, antigen-experienced human memory B cells moreover exhibited significantly increased TFEB expression, with IgD^+^ memory B cells exhibiting the highest TFEB levels of all investigated B cell subsets (Fig. 3j). Hence, our data establish TFEB as a cross-isotype BCR-distal nuclear effector, as well as an inter-species marker of B cells with antigenic experience.

### B cells employ non-canonical pathways of TFEB mobilization

In non-lymphoid cells, several conditions control the subcellular localization of TFEB by regulating its phosphorylation status, most notably via serine phosphorylation by the kinase mTOR. Additional kinases can generate complex TFEB phosphorylation patterns^30,31^. Phospho-TFEB is retained in the cytosol by associating with 14-3-3 proteins, masking the TFEB nuclear localization signal^32^. During nutrient starvation, the lysosomal release of Ca^2+^ ions activates the phosphatase calcineurin, which in turn dephosphorylates TFEB, resulting in the release of 14-3-3 and TFEB nuclear entry^31^.

**Fig. 4:**
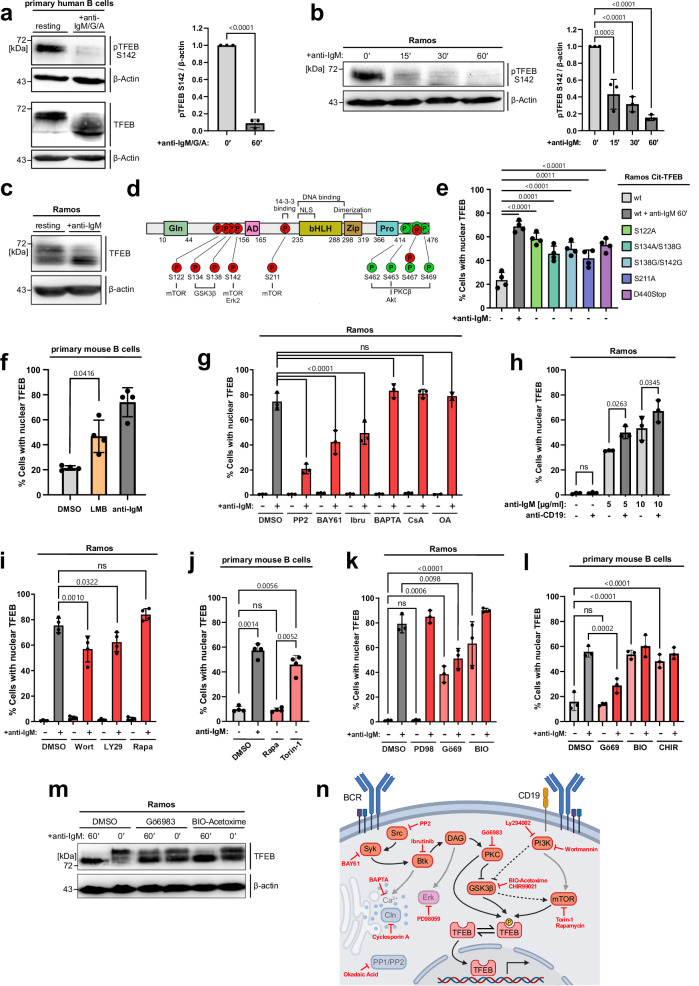
BCR signaling mobilizes TFEB through kinase inhibition. **a**–**c** Resting or BCR-stimulated primary human B cells from the blood of healthy donors (**a**) or Ramos B cells (**b**, **c**) were subjected to immunoblot analysis of total TFEB and phospho-S142 TFEB. Quantification of phospho-S142 TFEB in (**a**, **b**) was normalized to β-actin and is depicted as mean ± SD of *n* = 3 independent experiments. Statistical significance was computed using (**a**) an unpaired two-tailed Student’s t-test or (**b**) Tukey-corrected one-way ANOVA. **d** Schematic representation of reported TFEB phosphorylation sites (P) within individual TFEB domains indicated by GLN (glutamin-rich), AD (transcriptional activation), bHLH (basic helix-loop helix), Zip (leucine zipper), Pro, (proline-rich) and NLS (nuclear localization site). **e** Ramos transductants expressing TFEB variants were left untreated (−) or BCR-stimulated (+), and TFEB translocation was analyzed by imaging flow cytometry as described before. **f** CD19^+^ B cells of age-matched C57BL/6 mice were treated with DMSO, incubated with 20 nM leptomycin B (LMB) or BCR-stimulated for 60 min. **g** Wild-type Ramos B cells were left untreated (−) or BCR-activated (+, 60 min) in the presence of the following pharmacological agents: PP2, BAY61-3606 or ibrutinib (inhibiting Src, Syk or Btk, respectively), or the Ca^2+^ chelator BAPTA-AM, or cyclosporin A (calcineurin inhbitor) or okadaic acid (inhibitor of PP1 and PP2) and analyzed for TFEB translocation. **h** Wild-type Ramos B cells were treated with anti-BCR antibodies or 10 µg/ml anti-CD19 antibodies as indicated. **i**–**l** Nuclear translocation of TFEB in wild-type Ramos (**i** and **k**) or CD19^+^ splenic B cells of age-matched C57BL/6 mice (**j** and **l**) were left untreated (−) or BCR-activated (+, 60 min) in the presence of the following pharmacological inhibitors: Wortmannin, LY294002 (both PI3K), rapamycin (mTOR), torin-1 (ATP-competitive mTOR inhibitor), PD98059 (ERK), BIO-Acetoxime (GSK3β), CHIR99021 (GSK3β) or Gö6983 (PKC). Data is depicted as mean ± SD of *n* = 3 (**g**, **h**, **k** and **l**) or *n* = 4 (**e**, **f**, **i** and **j**) independent experiments. Statistical significances were computed using Tukey-corrected one-way ANOVA. **m** Immunoblot analysis of TFEB phosphorylation in Ramos B cells left untreated or BCR-activated in the presence of the indicated kinase inhibitors. **n** Schematic representation of the examined signaling network. This graphical overview was created with BioRender.com under a Creative Commons Attribution-NonCommercial-NoDerivs 4.0 international license. Source data are provided as a Source Data file.

To delineate the signaling pathway(s) that govern antigen-induced TFEB translocation in B cells, we first examined the phosphorylation of serine residue 142 (S142), a functionally relevant phospho-acceptor site ^26,27^ (Fig. 4a). In primary human B cells, S142 was constitutively phosphorylated and underwent robust dephosphorylation on BCR ligation together with additional phospho-serine residues, causing a marked shift in the electrophoretic mobility of TFEB (Fig. 4a), as reported for other cell types^33^. Ramos B cells recapitulated these phosphorylation patterns (Fig. 4b, c) and were chosen for investigating individual phosphorylation sites by mutational analysis. We generated Ramos B cell transductants expressing equal amounts of phosphorylation-deficient TFEB variants (Supplementary Fig. 3a), mimicking the dephosphorylation of single serine residues or that of a C-terminal serine cluster (Fig. 4d). Basically, all variants showed significant nuclear localization already in the absence of BCR ligation (Fig. 4e). However, none of them fully mimicked the nuclear positivity of wild-type TFEB found in activated B cells, suggesting a more complex regulation of TFEB nuclear residency. This conclusion was supported by a stimulation-independent nuclear accumulation of TFEB following treatment with leptomycin B (Fig. 4f and Supplementary Fig. 3b), an inhibitor of exportin 1-mediated nuclear export. These results point to a constant nuclear shuttling of TFEB, suggesting that it may have transcriptional functions also in resting B cells. This prompted us to identify TFEB-regulating B cell kinases and phosphatases. Pharmacological inhibition of the BCR-proximal kinases Src, Syk and Btk each diminished TFEB translocation, while inhibition of calcineurin or chelation of cytosolic Ca^2+^ showed no effect (Fig. 4g). Hence, the Ca^2+^ flux-inducing kinase axis Src-Syk-Btk promotes TFEB translocation, albeit independently of Ca^2+^ itself and the Ca^2+^ effector phosphatase calcineurin. Also, inhibition of the phosphatase families PP1 and PP2A failed to prevent the accumulation of nuclear TFEB. Since PP1 and PP2A together with calcineurin (PP2B) account for the majority of the serine/threonine phosphatase activity in live cells^34^, B-lymphoid regulation of TFEB seemed not to be controlled by mere activation of phosphatases but rather the inhibition of TFEB-phosphorylating kinases downstream of the BCR. Additional complexity was suggested by increased TFEB translocation following simultaneous stimulation of the BCR and its co-receptor CD19 (Fig. 4h). Indeed, inhibition of the CD19-proximal phosphatidyl-inositol-3 kinase (PI3K) dampened TFEB translocation (Fig. 4i). A known PI3K target is the AKT/mTOR axis^35^, and mTOR serves as ‘steady-state’ kinase in a number of cell types^36^. Canonical and non-canonical pathways of steady-state mTORC1 substrate phosphorylation exist^37^. Both pathways share the requirement of mTORC1 to be physically associated to the lysosomal membrane, and differ by their mode through which mTORC1 recruits its downstream targets. The allosteric mTORC1 inhibitor Rapamycin, which inhibits canonical mTORC1 substrate phosphorylation, had no effect on the subcellular localization of TFEB, neither in Ramos (Fig. 4i), nor in primary mouse B cells (Fig. 4j). However, treatment of B cells with Torin-1, an ATP-competitive inhibitor of both canonical and non-canonical mTORC1 activity, induced TFEB nuclear translocation to some extent in Ramos cells (Supplementary Fig. 3c), and even more pronounced in primary mouse B cells (Fig. 4j). Equal inhibition of tonic and BCR-induced canonical mTORC1 function by both inhibitors was confirmed by reduced phosphorylation of S6, a canonical target of mTORC1 activity (Supplementary Fig. 3d). Moreover, we examined TFEB-regulating kinases ERK, PKC and glycogen synthase kinase 3 (GSK3)^26,38,39^. Unlike ERK inhibition, PKC or GSK3 inhibition induced TFEB translocation in resting Ramos B cells, while inhibiting PKC also dampened its inducible nuclear entry (Fig. 4k). The role of GSK in controlling TFEB translocation was independently validated with an additional GSK inhibitor (CHIR99021) in Ramos B cells (Supplementary Fig. 3e) and also demonstrated in primary murine B cells (Fig. 4l). Consistently, inhibition of either GSK3 or PKC caused TFEB dephosphorylation (Fig. 4m).

Altogether, our data suggest that BCR and CD19 pathways, which are both triggered by antigen stimulation, cooperatively mobilize TFEB by inhibiting its baseline phosphorylation, shifting the dynamic equilibrium towards dephosphorylated TFEB and thereby, towards its nuclear translocation (Fig. 4n). BCR-induced production of diacylglycerol activates PKC, which retains TFEB in the cytosol by direct phosphorylation of TFEB, and indirectly, by phosphorylation-mediated inhibition of GSK3β, a known PKC target ^40^ and principal TFEB regulator kinase^41^. CD19 engagement further inhibits GSK3β, as a downstream target of the PI3K pathway, through phosphorylation^35^. Thus, GSK3β appears to constitute the point of convergence between BCR and CD19 pathways, which cooperatively control non-canonical TFEB phosphorylation, either by direct phosphorylation of TFEB, by regulating substrate availability of TFEB towards mTOR, or by both mechanisms.

### TFEB shapes the transcriptional landscape of GC B cells

**Fig. 5:**
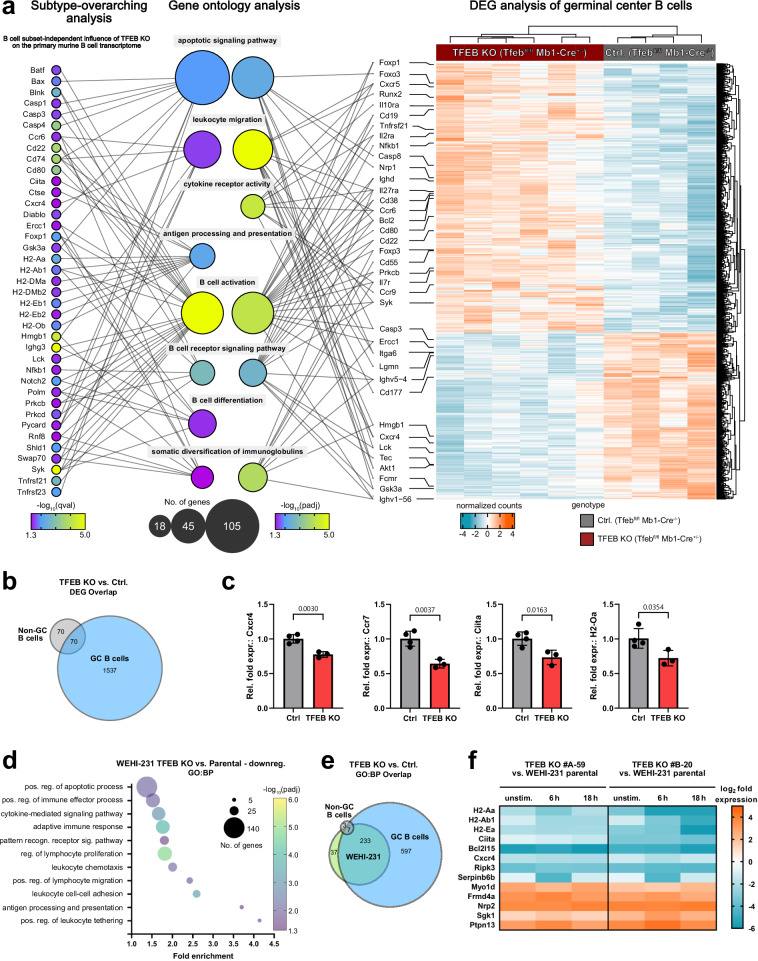
TFEB shapes the transcriptional landscape of GC B cells. **a**–**e** Splenocytes from TFEB-deficient C57BL/6 mice (*n* = 6) and control littermates (*n* = 4) were sorted into B220^+^Fas^+^GL7^+^ germinal center (GC) and B220^+^Fas^-^GL7^-^ non-GC B cells and subjected to bulk RNA sequencing. **a** B cell subtype-overarching analysis shows the transcriptional deregulation explained by TFEB’s absence across non-GC and GC subsets. DEGs were defined by a qval< 0.05, with selected DEGs shown on the left. Statistical significances were computed using likelihood-ratio tests, corrected for multiple testing via Benjamini & Hochberg’s method. The heatmap on the right shows all > 1600 DEGs (padj< 0.05) in TFEB-negative GC B cells and their clustering among genotypes and samples. Statistical significances were computed using Wald tests, adjusted by Benjamini & Hochberg’s procedure. Selected DEGs found in either analysis were mapped onto relevant “biological process” GO terms (GO:BP, middle). GO enrichment and the number of DEGs per term are reflected in the respective bubble’s color and size. Selected target genes are connected via line to their respective GO terms. **b** Venn diagram of DEGs between TFEB-deficient GC and non-GC B cells. **c** FACS-sorted non-GC B cells from TFEB KO (*n* = 4) and control littermates (*n* = 3) were subjected to qRT-PCR analysis. Normalized expression of selected genes is depicted as mean ± SD. Statistical significances were computed using unpaired two-sided Student’s t-tests. **d**–**f** Independent TFEB-deficient WEHI-231 clones (#A-59 and #B-20) and parental cells were left untreated or BCR-stimulated for 6 or 18 h and subjected to RNA sequencing. Data were derived from *n* = 2 independent experiments using two mutant clones. DEGs were defined by an FDR < 0.01 and a log_2_ fold change of > 1 or < −1 and were subjected to gene ontology analysis (‘GOrilla’). **d** Selection of highly enriched GO terms. The number of genes per term is illustrated by bubble size, while adjusted FDR values are represented through a color gradient. **e** Venn diagram of significantly enriched GO:BP terms between TFEB-depleted GC B cells, non-GC B cells and WEHI-231 knockouts. **f** Heatmap depicting log_2_ fold changes of selected DEGs with immunological relevance for TFEB mutant clones versus parental WEHI-231 cells. Source data are provided as a Source Data file.

Having demonstrated TFEB’s activation in response to antigenic stimulation, we aimed to explore B-lymphoid TFEB functions in naïve and antigen-experienced GC cells. To that end, we generated a conditional mouse mutant with B cell-specific ablation of TFEB expression by breeding mice harboring a floxed TFEB allele (TFEB^fl^) ^26^ with the Mb1-Cre deleter strain (Supplementary Fig. 4a). Non-GC (Fas^−^GL7^−^) and GC B cells (Fas^+^GL7^+^) were isolated from splenocytes of TFEB mutants (TFEB^fl/fl^ Mb1-Cre^+/−^) or control littermates (TFEB^fl/fl^ Mb1-Cre^−/−^) by fluorescence-activated cell sorting (Supplementary Fig. 4b) and subjected to bulk RNA sequencing analysis (Fig. 5a, b). TFEB-deficient non-GC B cells, which primarily consist of naïve B cells without antigen experience displayed a moderate number of 140 differentially expressed genes (DEGs) defined by an adjusted *p* < 0.05 (Fig. 5b), consistent with a putative role of TFEB in resting B cells. A priori, DEGs do not necessarily represent direct TFEB targets that typically have a TFEB consensus recognition motif in their promotor region called CLEAR for ‘coordinated lysosomal expression and regulation’^42^. Indirect modes of deregulated gene activities caused by transcriptional regulators being themselves a TFEB target are likely to exist and might especially apply to those genes that were transcriptionally up-regulated upon loss of TFEB. A more fundamental impact of TFEB was revealed for the transcriptional profile of the GC population, which represents antigen-experienced B cells as cognate BCR ligation is a prerequisite for GC entry^43^. TFEB deficiency in GC B cells yielded > 1600 DEGs, which is more than 10 times the number found in non-GC B cells and thus indicates a dominant role of TFEB in the transcriptional reprogramming of BCR-activated cells.

We first conducted a subtype-overarching transcriptome analysis of TFEB-negative versus littermate-derived control B cells (Fig. 5a, left). The depicted DEGs were chosen for their functional association to selected significantly enriched GO terms of central importance to B cell immunity, including ‘B cell activation’ and ‘differentiation’ (Fig. 5a, middle, Supplementary Fig. 4c). Among these GO-defining DEGs (qval< 0.05) were direct components of the ‘BCR signaling pathway’ (Syk, Prkcb, Gsk3a, Nfkb1). Strikingly, among the deregulated transcripts were several modulators of ‘leukocyte migration’ (Cd74, Cxcr4, Ccr6), as well as MHC II family members (H2-Aa, H2-Eb1, H2-Oa) and their transactivator Ciita, key players of the ‘antigen processing and presentation’ machinery. The presence and/or BCR-induced expressional increase of these surface proteins are pivotal for full B cell activation by co-stimulatory signals^8^. TFEB-controlled transcription of selected genes with central importance for B cell differentiation (Cxcr4, Ccr7, H2-Oa and Ciita) was further verified via qPCR (Fig. 5c). Notwithstanding, the B-lymphoid transcriptome of TFEB however also encompassed cell death-associated elements of the ‘apoptotic signaling pathway’, exemplified by genes encoding members of the caspase family, as well as the pro-apoptotic Bcl-2 family member Bax and its activator Pykard.

Next, we aimed to explore TFEB’s role at the interface of B cell tolerance induction versus B cell activation during the GC reaction where apoptotic BCR signals become integrated with T_FH_-provided CD40 survival signals^44^. We therefore applied GO analysis to the DEGs (padj< 0.05) found in TFEB-deficient GC B cells (Fig. 5a, right, Supplementary Fig. 4d). Again, multiple regulators of ‘cell migration’ (e.g., Lgmn, Runx2) and ‘cytokine receptor activity’ (e.g., Cxcr4, Cxcr5, Ccr9, Cd69, Il7r, Il27ra, S1pr2, Tnfrsf2 were broadly affected. While they all promote the development of B cells and their homing to secondary lymphoid organs, Cxcr4, Cxcr5, Cd69, and S1pr2 have been directly linked to the GC reaction^45–47^. Also, the BCR itself (Ighd), as well as associated signaling elements (e.g., Syk, Prkcb, Nfkb1, Cd19) were upregulated in TFEB-deficient GC B cells, suggesting a negative feedback loop or a compensatory mechanism. Moreover, expression of additional drivers of B cell differentiation, like central genes governing ‘somatic diversification of immunoglobulins’ (e.g., Ercc1, Hmgb1) as well as multiple regulators of ‘cell cycle progression’ (e.g., Cdk1, Aurka, Cdca8, Cdkn1b), were diminished in TFEB-deficient GC B lymphocytes. Nevertheless, further significant enrichment of GO terms related to apoptotic cell death was likewise detected, with downregulation of the central pro-apoptotic executor caspase-3 (Casp3) and upregulation of anti-apoptotic Bcl-2 in TFEB-negative GC B cells. TFEB thus appears to promote both B cell activation and apoptosis, indicating a decisive role for fate determination of activated B cells. Notably, gene ontology analyses did not reveal a significant overlap with autophagy-related cell functions. Instead, transcriptome-wide analysis of our conditional mouse mutant suggests a key role of TFEB in shaping the B-lymphoid transcriptional profile, which appears to be of particular importance for conducting fate decisions within the GC.

To further corroborate B-lymphoid TFEB functions, we generated TFEB-deficient mutants of murine WEHI-231 and human Ramos B cells (Supplementary Fig. 5). Comprehensive RNA sequencing analyzes of wild-type and TFEB-deficient B cells revealed the B-lymphoid transcriptional activities of TFEB under resting and stimulating conditions. WEHI-231 and Ramos parental cells and two independently generated TFEB mutants were each either left untreated or BCR-stimulated for 6 or 18 h, and subjected to transcriptome analyzes. Differentially regulated genes were defined by transcripts with an FDR < 0.01 and a log_2_-fold change of > 1 or < −1 (Supplementary Fig. 6a, b). In accordance with the TFEB-dependent transcripts discovered in primary mouse B cells, gene ontology analysis of biological processes (Fig. 5d, Supplementary Fig. 6c), of molecular functions or of cellular components (Supplementary Fig. 6e–h) did not reveal a significant overlap with autophagy-related functions, neither in the transcriptome of human nor in that of murine B cells and despite the fact that our approach encompassed resting and stimulating conditions. Consistently, genes controlling central functions of B cell activation, such as proliferation, migration, signaling and antigen presentation, but also mediators of apoptotic cell death were once more deregulated, instead (Fig. 5d, Supplementary Fig. 6c). Altogether, more than 25% of the TFEB-dependent transcripts found in stimulated WEHI-231 cells were also found in primary mouse GC B cells. This overlap resulted in a striking functional overlap of over 85% of the significantly enriched “biological function” Gene Ontology (GO) terms (padj< 0.05) between stimulated WEHI-231 and mouse GC B cells (Fig. 5e).

**Fig. 6:**
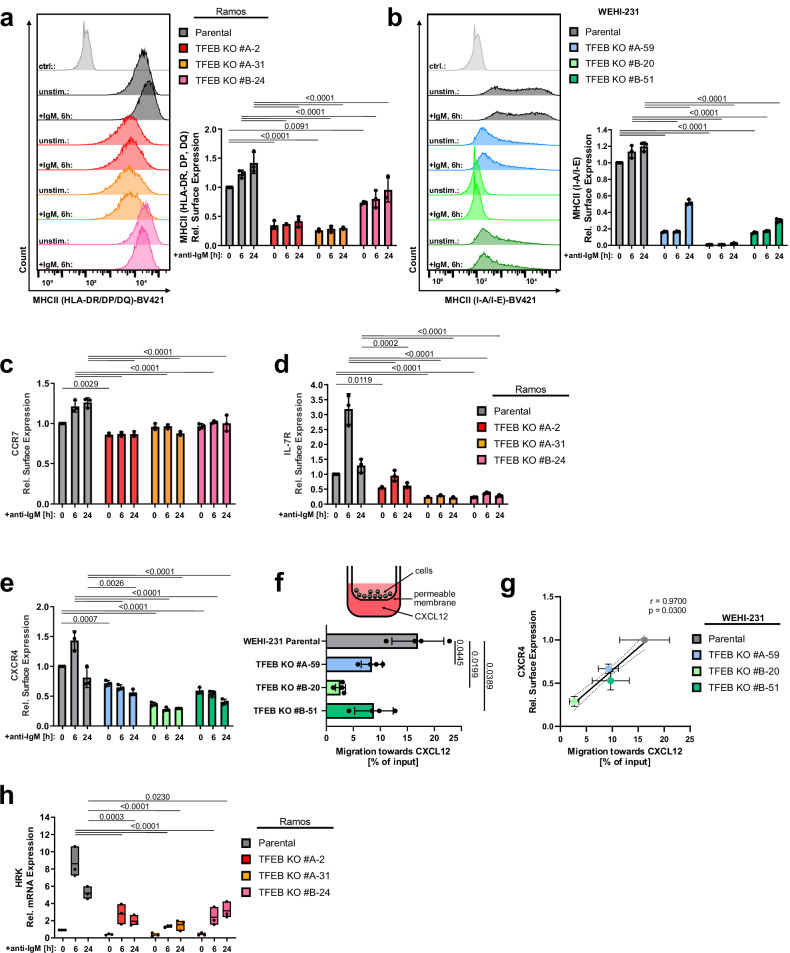
TFEB governs B cell responsiveness to co-stimulatory signals. Wild-type or three independently generated TFEB mutants of either Ramos (**a**, **c**, and **h**) or WEHI-231 cells (**b** and **e**) were left untreated or BCR-stimulated for the indicated time periods. Subsequently, cell surface expression of MHC class II proteins (**a**, **b**), the cytokine receptors CCR7 (**c**), IL-7 R (**d**) and CXCR4 (**e**) was analyzed by flow cytometry. **f** The migratory capability of wild-type or TFEB mutant WEHI-231 cells towards CXCL12 was measured through transwell migration assays. Data in (**a**–**f**) are presented as mean ± SD of *n* = 4 independent experiments. Statistical significances were computed using one-way ANOVA and corrected for multiple testing via Dunnett’s method. **g** The amount of surface CXCR4 expression measured in (**e**) was plotted against the migration efficiency towards CXCL12 depicted in (**f**). The linear relationship between these parameters was analyzed using Pearson’s correlation. Statistical significance is depicted as correlation coefficient and the corresponding two-sided *p* value. Linear regression is shown as solid line and the 95% CI is marked with dotted curves. **h** HRK relative mRNA expression was quantified by qRT-PCR using the ΔΔC_T_-method. Data is presented as mean of *n* = 3 independent experiments (with the box depicting min, max and the mean values). Source data are provided as a Source Data file.

Prominent examples of DEGs in TFEB-negative cell lines are MHC class II molecules and their primary transcriptional activator Ciita (Fig. 5f, Supplementary Fig. 6d), which enable antigen presentation by primed B cells towards cognate T_FH_ cells^11,12^. As shown in primary murine B cells, expression of MHC II proteins was markedly deregulated in TFEB-deficient WEHI-231 and Ramos B cells, which also translated to their severely compromised constitutive and inducible surface deposition (Fig. 6a, b). The chemokine receptors Ccr7 and Cxcr4 govern T-B cell collaboration more indirectly by guiding immune cells to and within lymphoid organs. Specifically, BCR-induced Ccr7 steers B cells towards the T cell zone where they seek cognate T cell help at the T cell:B cell border, while Cxcr4 controls entry into the GC dark zone^48,49^. Other cytokine receptors, like Il-7r, directly promote B cell development and differentiation^50^. The steady-state levels of Cxcr4, Ccr7 and Il-7r were reduced on TFEB-deficient WEHI-231 and Ramos B cell mutants, respectively, and the stimulation-induced increase was abolished on both the transcriptional (Fig. 5f, Supplementary Fig. 6d) and cell surface expression level (Fig. 6c–e). Moreover, migration towards Cxcl12, the chemokine ligand of Cxcr4, was severely diminished in all three TFEB-negative mutant variants (Fig. 6f, g). In summary, TFEB deficiency compromises the ability of primed B cells to receive and process extracellular signals that are necessary for trafficking to and interacting with T_FH_ cells. Additionally, loss of TFEB severely alleviated the BCR-controlled biogenesis of lysosomes and mitochondria (Supplementary Fig. 7a–d), which is necessary to effectively initiate antigen processing and presentation^51^, and allows activated cells to adapt to altered energy requirements^15^, respectively.

Once more, the transcriptome analysis of TFEB-deficient cells revealed cell death-associated elements exemplified by genes encoding members of the Bcl-2 homology (BH) 3 family of pro-apoptotic regulators (Hrk, Bcl2l15) (Fig. 5f and Supplementary Fig. 6d). Strongly reduced Harakiri (HRK) expression in response to BCR-stimulation in TFEB-deficient Ramos mutants was further validated through qPCR, indicating a markedly reduced susceptibility to apoptosis (Fig. 6h). Collectively, the data suggest that the transcriptional activity of TFEB renders primed B cells capable of finding and receiving T cell help within the GC, but seems to simultaneously promote B cell death in the absence of subsequent rescue signals.

### TFEB integrates BCR-induced cell death with CD40 survival signals

**Fig. 7:**
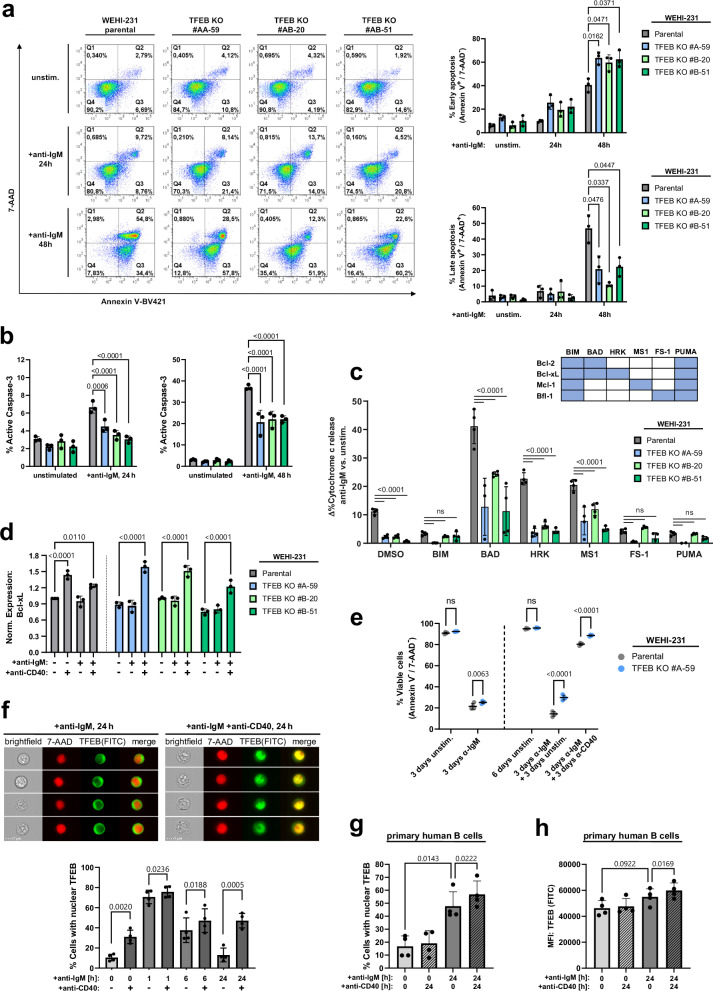
TFEB balances pro-apoptotic BCR with CD40 rescue signals. **a** Parental and TFEB-mutant WEHI-231 B cells were left untreated or BCR-stimulated for the indicated periods. Flow cytometric co-staining with Annexin V-BV421 and 7-AAD was used to detect early (Annexin V^+^/7-AAD^-^) and late apoptosis (Annexin V^+^/7-AAD^+^). **b** Flow cytometry analysis of active caspase-3 in WEHI-231 cells treated as described above. Data in (**a**, **b**) are depicted as mean percentage±SD of gated cells from *n* = 3 independent experiments. Significances were computed using Dunnett-corrected two-way ANOVA. **c** BH3 profiling of TFEB-depleted and parental WEHI-231 cells using the indicated BH3-agonistic peptides. Binding specificities towards Bcl-2 family proteins are indicated in the corresponding matrix. Cytochrome c release was monitored for resting cells or after 6 h of BCR ligation by flow cytometry. BCR-induced sensitization is presented as mean ± SD percent difference (Δ%) between *n* = 4 technical replicates of stimulated versus unstimulated cells. Depicted data are representative of *n* = 3 biological replicates. Statistical significances were calculated using Tukey-corrected one-way ANOVA. **d** WEHI-231 cells were incubated with the indicated combinations of anti-BCR and anti-CD40 antibodies for 6 h. Intracellular Bcl-xL expression was assessed by flow cytometry and is depicted as MFI, normalized to unstimulated parental cells. **e** Parental WEHI-231 and TFEB KO #A-59 cells were left untreated or stimulated, as indicated. Medium was changed on day 3, and cells were left untreated or were rescued with anti-CD40 until day 6. On day 3 and day 6, cells were incubated with Annexin V/7-AAD to access viability, as defined by a double negative staining. **f** WEHI-231 cells were BCR-stimulated for the indicated time periods in the presence or absence of CD40 ligation. Imaging flow cytometry-derived representative images and nuclear translocation of TFEB as percentages of cells with a TFEB/7-AAD similarity score ≥ 1. **g**, **h** Primary peripheral human CD19^+^ B cells were left untreated or BCR-stimulated for 24 h with or without CD40 ligation. Nuclear translocation (**g**) and expression (**h**) of TFEB was measured by imaging flow cytometry. Data shown in (**d**–**h**) is presented as mean ± SD of *n* = 3 (**d**) or *n* = 4 (**e**–**h**) independent experiments. Statistical significances were calculated using Tukey-corrected one-way ANOVA. Source data are provided as a Source Data file.

Given the impaired up-regulation of HRK and Bfk (Bcl2l15) in TFEB-deficient Ramos and WEHI-231 B cells, respectively, we tested whether TFEB impacts on activation-induced cell death^52,53^. We thus monitored early and late apoptotic stages in BCR-activated WEHI-231 cells (Fig. 7a), a model system to study BCR-induced cell death in mature B cells^54^. Parental WEHI-231 cells entered the early phase of apoptosis after 24 h of BCR engagement, and the majority of cells advanced to the late apoptotic phase after 48 h. By contrast, TFEB-deficient mutants displayed an arrest in the early phase of apoptosis demonstrating that TFEB expression promotes the completion of apoptosis. Accordingly, TFEB-deficient cells exhibited significantly reduced caspase-3 activity after 24 h and 48 h of BCR stimulation (Fig. 7b). Cells that survived BCR ligation still held the potency for clonogenic colony formation, which was significantly enhanced in TFEB-deficient cells (Supplementary Fig. 8b).

Next, we determined the role of pro-apoptotic Bcl-2 family members in driving late phase apoptosis by using BH3 profiling^55^. Briefly, BH3 profiling is a peptide-based method to reveal the individual contribution of BH3 domain-containing activators of apoptosis (so-called BH3-only proteins, schematically depicted in Supplementary Fig. 8c) to the cell-intrinsic pathway of apoptosis that is characterized by the release of cytochrome c from mitochondria. Technically, cells receive agonistic peptide mimetics of BH3-only proteins that then sensitize these cells to intrinsic apoptosis by counteracting individual members of the pro-survival Bcl-2 members (Fig. 7c, table). BH3-sensitized wild-type and TFEB-deficient WEHI-231 cells were left untreated or BCR-stimulated, and subsequently, cytochrome c release was monitored by flow cytometry. Apoptotic sensitization scores were depicted as the difference of mean percentages (Δ%) of cytochrome c release between stimulated and non-stimulated cells (Fig. 7c, Supplementary Fig. 8a). The selective inhibition of pro-survival proteins by mimetics of BAD, HRK and MS1 sensitized WEHI-231 cells to BCR-induced cell death in a dose-dependent manner. Intriguingly, the HRK and BAD mimetics appeared to be the most effective sensitizers for BCR-induced apoptosis, given the most pronounced sensitization index was detected for HRK and the overall highestcytochrome c release was induced by BAD. While HRK only inhibits Bcl-xL, BAD additionally acts on Bcl-2, which indicates that BCR-induced apoptosis execution is achieved through targeting Bcl-xL, Bcl-2 or both. Altogether, TFEB-induced up-regulation of the BH3-only member HRK and consequent inhibition of the Bcl-2 family protein Bcl-xL provides a direct mechanistic link between BCR ligation and B cell death by apoptosis.

Consistent with previous reports^56,57^, the expression of Bcl-xL increased following treatment of WEHI-231 cells with anti-CD40 antibodies alone or in combination with anti-BCR antibodies but not on mere BCR ligation (Fig. 7d). This induction of expression was also observed in all TFEB-negative variants revealing a TFEB-independent mechanism of CD40-mediated apoptosis prevention. The augmented Bcl-xL expression was associated with three intriguing observations. Firstly, and in line with the literature, CD40 ligation salvaged wild-type WEHI-231 cells from BCR-triggered early and late apoptosis (Supplementary Fig. 8d). Secondly, loss of TFEB and the resulting delay in apoptosis improved the survival of BCR-activated cells both in the absence or presence of CD40 rescue signals (Fig. 7e, Supplementary Fig. 8e). Thirdly, while simultaneously inhibiting TFEB-induced apoptosis by upregulation of Bcl-xL, CD40 engagement turned out to be a potent TFEB activator as revealed by imaging flow cytometry of WEHI-231 cells under various stimulation conditions (Fig. 7f). Compared to resting cells, mere CD40 ligation initiated robust nuclear translocation of TFEB, albeit less efficient than BCR ligation. In the absence of CD40 signaling, TFEB nuclear positivity returned to the baseline level of resting cells during continued BCR ligation. On the contrary, the combined stimulation of BCR with CD40 effectively decelerated and eventually blocked the decline so that the nuclear TFEB level observed after 6 h was maintained even after 24 h of co-stimulation. CD40-mediated amplification of TFEB translocation was also observed in human Ramos B cells (Supplementary Fig. 8f) as well as in primary human B cells (Fig. 7g). Furthermore, CD40 not only increased nuclear TFEB in primary B cells, but also enhanced TFEB expression after 24 h of co-stimulation (Fig. 7h). CD40-induced nuclear accumulation of TFEB was abolished in the presence of wortmannin, indicating a role of PI3K signaling in CD40-controlled TFEB regulation (Supplementary Fig. 8g). In conclusion, CD40 co-stimulation sustains the nuclear residency of TFEB that is accompanied by a TFEB-independent heightened expression of Bcl-xL. As a net result, balancing HRK expression with Bcl-xL in WEHI-231 cells halts the TFEB-driven progression of cell-intrinsic apoptosis. Altogether, our results unveil the interplay between pro- and anti-apoptotic Bcl-2 family members as a common signal axis downstream of BCR and CD40 signaling.

### TFEB orchestrates GC fate decisions

**Fig. 8:**
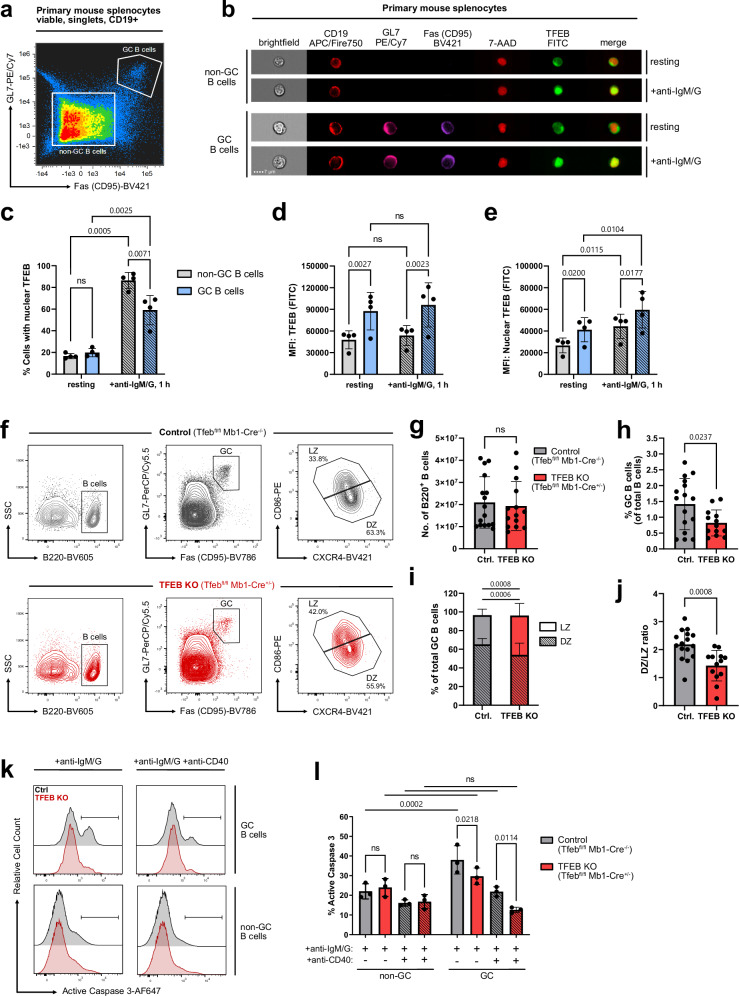
TFEB promotes antigen-induced apoptosis in GC B cells. **a**–**e** Mouse splenocytes were left untreated or stimulated with anti-IgM/G for 60 min and analyzed for TFEB expression and subcellular distribution by imaging flow cytometry. **a** Staining strategy to distinguish germinal center and non-GC B cells from total splenocytes. GC B cells were gated as CD19^+^Fas^+^GL7^+^, whereas non-GC B cells were defined as CD19^+^Fas^-^GL7^-^ (gating depicted in Supplementary Fig. 9a). **b** Representative multi-channel images of individual resting and BCR-stimulated germinal center and non-GC B cells. **c** Percentage of cells with translocated TFEB, as defined by TFEB/7-AAD similarity score ≥ 1. **d** Mean fluorescence intensity of TFEB. **e** Mean fluorescence intensity of TFEB within the nucleus. Data are depicted as mean ± SD of *n* = 4 littermates. Statistical significances were computed using RM two-way ANOVA, corrected for multiple testing via Tukey’s method. **f**–**j** Flow cytometric analyzes of GC subsets in B cell-conditional TFEB mutant mice (*n* = 13) and control littermates (*n* = 16). **f** GC B cells gated as B220^+^Fas^+^GL7^+^. were further distinguished in LZ and DZ GC B cells by their expression of CD86 and CXCR4. **g** Absolute number of splenic B220^+^ B cells. **h** Percentage of B220^+^Fas^+^GL7^+^ GC B cells among total B220^+^ B cells. **i** Percentage of LZ and DZ GC B cells among total GC B cells. **j** Ratio of LZ and DZ GC B cells. Data in (**g**–**j**) are depicted as mean ± SD. Statistical significances were computed using Šidák-corrected RM two-way ANOVA (**i**) or two-tailed Student’s t-test (**g**–**j**). **k**, **l** Splenocytes of TFEB-depleted and control mice were isolated and stimulated with anti-IgM/G and co-stimulated with CD40 for 18 h, as indicated. CD19^+^ B cells were further subgated into germinal center (CD19^+^Fas^+^GL7^+^) and non-GC B cells (CD19^+^Fas^-^GL7^-^; gating depicted in Supplementary Fig. 9b) and co-stained with anti-active caspase-3-AF647 and Zombie Green dye. **k** Selected histograms depicting active caspase-3 fluorescence intensity. **l** Proportions of active caspase-3-positive B cells among GC or non-GC B cells, depicted as mean ± SD of *n* = 3 independent experiments. Statistical significances were computed using RM two-way ANOVA and Fisher’s LSD test. Source data are provided as a Source Data file.

Since GC B cells have already undergone at least one instance of antigenic stimulation, we imaged GC B cells for remaining traces of TFEB activation and furthermore assessed whether they are prone to effective (re-)stimulation via the BCR. Indeed, despite a normal baseline level of nuclear TFEB (Fig. 8a–c, and Supplementary Fig. 9a), the overall expression of TFEB in murine GC B cells was almost doubled in comparison to non-GC B cells (Fig. 8d). Consequently, the amount of TFEB in the nuclei of resting GC B cells was strongly elevated and reached a level that was observed in non-GC B cells only after BCR engagement (Fig. 8e). However, GC B cells were still able to recruit even more TFEB to their nuclei on BCR activation. In total, relatively few (~60%) of GC B cells responded with nuclear translocation of TFEB (Fig. 8c), which may be due to generally reduced BCR signaling in GC B cells^58^. These data nonetheless indicate that the increase in TFEB expression is imprinted in GC B cells, thus establishing a TFEB-driven transcriptional program that primes the B cell for consecutive stimulation events. This conclusion is consistent with our finding of increased nuclear steady-state levels of TFEB in murine and human memory B cell populations (see Fig 3).

During the GC reaction, primed B cells cycle between the GC dark zone (DZ) and light zone (LZ), in which they undergo proliferation and somatic mutation, or selection for proper antigen binding and co-stimulation, respectively^59^. We therefore investigated the effect of TFEB deletion on the composition of GC B cell subsets (Fig. 8f). While TFEB-deficiency did not alter the total number of splenic B cells (Fig. 8g), the frequency of GC B cells was markedly reduced (Fig. 8h), further affirming a crucial involvement of TFEB in the course of the GC reaction.

Strikingly, conditional TFEB mutant animals showed a significantly increased fraction of LZ and a proportional decrease of DZ B cell frequencies among GC B cells (Fig. 8i), resulting in an abnormal DZ/LZ B cell ratio (Fig. 8j). Accordingly, we found prominent downregulation of the DZ marker CXCR4 in GC B cells, as well as reduced expression of transcripts related to cell cycle progression and proliferation (see Fig. 5a), processes predominantly occurring in the DZ.

Finally, we aimed to test TFEB’s role in promoting antigen-induced apoptosis in GC B cells, as suggested by the downregulation of pro-apoptotic Bax and upregulation of anti-apoptotic mediator Bcl-2 in TFEB mutant mice (see Fig. 5a). To this end, GC and non-GC B cells (Supplementary Fig. 9b) were assessed for their susceptibility to undergo BCR-induced cell death by monitoring active caspase-3-positivity (Fig. 8k, l) and Zombie Green positivity (Supplementary Fig. 9c). In accordance with the literature, the overall apoptosis rate was higher in GC than in non-GC B cells. However, TFEB-deficient splenic GC B cells exhibited a severely diminished pro-apoptotic response to BCR stimulation, both in the presence, as well as in the absence of anti-CD40 rescue signals (Fig. 8k, l), indicating a pro-apoptotic role of TFEB during the GC reaction. In summary, TFEB executes B-lymphoid fate decisions through the genetic reprogramming of antigen-experienced GC B cells.

## Discussion

**Fig. 9:**
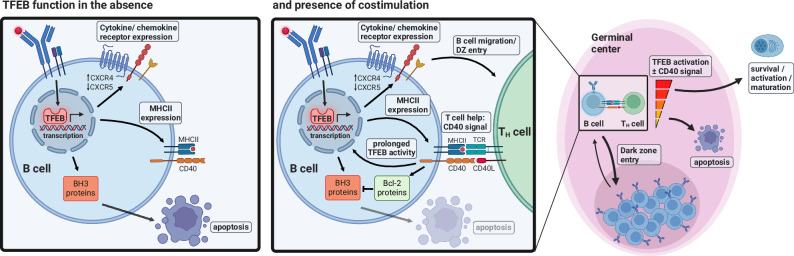
Proposed mechanisms by which TFEB governs B-lymphoid fate decisions. Antigenic stimulation induces nuclear translocation of TFEB. In the absence of T cell help, TFEB transcriptional activity leads to death-by-neglect through upregulation of pro-apoptotic proteins (e.g., BH3-only proteins, caspase-3). Concomitantly, TFEB arranges for a possible rescue by promotion of B cell migration to lymph follicles and GC entry (e.g. via regulation of CXCR4 and CXCR5) as well as induction of antigen presentation via MHC II. In the presence of T-lymphoid co-stimulation, CD40 rescue prevents TFEB-driven apoptosis by upregulation of Bcl-2 family proteins. CD40 signaling concurrently prolongs TFEB nuclear activity, further enhancing TFEB’s stimulatory influence on B cells, leading to further activation and maturation through DZ (re-)entry. This graphical summary was created with BioRender.com under a Creative Commons Attribution-NonCommercial-NoDerivs 4.0 international license.

The two-signals requirement of B cell activation represents a well-established principle of humoral immunity and provides a mechanistic basis for the maintenance of humoral immune tolerance to self-antigens. However, the computation of antigen-mediated B cell priming with secondary co-stimulation remained elusive. Here, we identified TFEB as a transcriptional signal integrator that commits primed B cells to cell-intrinsic apoptosis via transcriptional modulation of Bcl-2 family proteins and their mitochondrial regulators, and yet, simultaneously, induces the expression of a potent toolkit for antigen-primed B cells to receive co-stimulatory help. Specifically, TFEB-driven organelle biogenesis and regulation of cytokine receptors supports cellular activation and differentiation, and moreover facilitates migration to lymphatic follicles and GC entry. Indeed, TFEB’s dual role of promoting both cell death and priming for co-stimulation culminates during the GC reaction (Fig. 9). GC B cells proliferate and undergo antigen receptor diversification in the DZ, while in the LZ the cells are tested for BCR affinity. TFEB maintains the balance of DZ and LZ GC B cells, probably by regulating expression of the key GC homing receptors CXCR4 and CXCR5. In the absence of TFEB, GC B cells express less CXCR4, which usually guides the cells to the dark zone^46^. By contrast, TFEB-deficient GC B cells showed upregulation expression of CXCR5, which guides the cells to the LZ. Consequently, in conditional TFEB-deficient mice the DZ/LZ ratio was skewed towards more LZ cells. In the DZ, TFEB supports the expression of genes that nurture B cell proliferation and antigen receptor diversification. Yet in the LZ, TFEB drives Bcl-2 family-mediated apoptosis of B cells devoid of cognate T cell help, but concurrently supports B cell-T_FH_ cell interactions by promoting antigen presentation via MHC II. The resulting T_FH_-provided CD40 signal does not merely rescue antigen-primed B cells from TFEB-promoted apoptosis, but fosters TFEB’s stimulatory potential even further by prolonging its nuclear localization. BCR-induced TFEB hence commits GC B cells to death-by-neglect in the absence of T cell help, however concurrently promotes the reception of cognate T-lymphoid rescue and activation signals, leading to further maturation within the DZ. In TFEB-deficient mice, GC fate consequently appears to be less strictly enforced, leading to a decline of GC B cell apoptosis in the absence of T_FH_ aid. Collectively, by integration of antigenic signaling with CD40 rescue, TFEB-driven transcriptional reprogramming ultimately steers B-lymphoid fate decisions towards either apoptosis in the absence, or B cell activation and maturation in the presence of co-stimulation. The dual role of TFEB in the decisive interplay between tolerance induction, activation and differentiation explains the apparent contradictory nature of BCR engagement being likewise essential for the elimination as well as for the activation of B cells.

TFEB was initially identified by binding to Ig-heavy chain enhancer elements called E-box sequences^60^. In non-lymphoid cells, TFEB coordinates a transcriptional program driving autophagy and lysosomal biogenesis^20^. In the absence of appropriate stimuli, mTORC1 phosphorylates and thereby retains TFEB in the cytosol^26,32^, while the dephosphorylation by calcineurin represents a common trigger of TFEB activation. As shown here, antigen-primed B cells employ a non-canonical mode of TFEB regulation that is characterized by the inhibition of GSK3β through a concerted action of BCR and CD19. The contribution of both non-canonical mTORC1 and GSK3β signals in the regulation of TFEB and the convergence of these pathways in B cells is intriguing and will be an important basis for further studies.

Our finding of TFEB mobilization being a hallmark of primary B cell activation was fostered by two technical advances. First, we established a proteomic identification method for signal elements that undergo a stimulation-dependent nuclear translocation. Second, imaging flow cytometry allowed for the visualization of that process with statistical significance. The combination of the two approaches revealed that the BCR-induced nuclear translocation and upregulated expression of TFEB is (i) conserved between human and murine B cells, (ii) common to different B cell developmental stages, (iii) more rapid and efficient than any other known shuttling element including NF-κB and NFAT1, (iv) crucial for fate choices in the course of the GC reaction and (v) amplified in as well as a constitutive feature of antigen-experienced GC and memory B cell subpopulations. The latter finding is of particular interest because a specific transcriptional activity that characterizes memory B cell subsets has not yet been identified. Up to 20% of unswitched and switched human CD27^+^ memory B cells displayed nuclear TFEB even in absence of ex vivo stimulation, which is paralleled by a markedly increased TFEB expression. Hence, a permanent or periodic nuclear residency of TFEB, as well as a boosted recall response fueled by elevated TFEB expression, may be characteristic properties of memory B cell subsets. Likewise, the markedly elevated TFEB nuclear levels in murine GC B cells indicate that persistent upregulation of TFEB is a cross-species hallmark of antigen-experienced B lymphocytes.

It was peculiar not to find autophagic effectors in our transcriptome analysis. However, it is well possible that TFEB activity governs autophagic processes during B cell developmental stages or under conditions others than those investigated here. For example, TFEB might control the longevity of memory B cells in agreement with the emerging role of autophagy on cell survival^61^. Indeed, TFEB activation was shown to rejuvenate aged B cells by reversing senescence and restoring immune function^62^. These findings are in concordance with our hypothesis that a permanent nuclear residency of TFEB contributes to the homeostasis of the human memory B cell compartment. While autophagy is increasingly being viewed as a prominent player in inflammation and inflammation-mediated diseases^63^, immune-specific functions of TFEB have also been described for macrophages and dendritic cells and autophagic activity is reported to modulate antigen cross-presentation^64–66^. In T cells, inactivation of the MiTF family members TFEB and TFE3 diminishes expression of CD40L, resulting in hyper IgM syndrome due to impaired T_FH_-B cell help^67^. TFEB moreover antagonizes malignant metabolic adaptation in Myc-driven B cell lymphomas, thereby suppressing uncontrolled cell growth^68^. Of note, B cells also express TFE3, which might conduct autophagic functions in addition to or even more dominantly than TFEB. Autophagosome formation upon BCR stimulation has been described by the group of Tsubata^69,70^. The same group also showed that CD40 ligation rescues BCR-induced apoptotic B cell death ^71^ by up-regulating the expression of Bcl-xL^56,57^. Intensive signal crosstalk between autophagy and especially the late stage of apoptosis exists^61^.

Our data described herein reveal another twist to this network. The master regulator of autophagy, TFEB, acts as a switch regulatory factor at the crossroads of B cell fate decisions during primary antigen encounters and secondary co-stimulation. The former leads to B cell apoptosis by default because a concerted action of the BCR and CD19 governs a B-lymphoid TFEB nuclear translocation mechanism directing the expression of mitochondrial effectors of cell death. Concomitantly, TFEB arranges for a possible salvage pathway by inducing B cell antigen presentation as well as the expression of sensors promoting migration to lymph node follicles and GC entry, where they eventually meet with cognate T_FH_ cells, ultimately providing CD40 rescue. As TFEB participates in all of these B cell fate decisions, further deciphering of the underlying mechanisms might contribute to a better understanding of autoimmune responses and also to improving the development of vaccines with optimized efficacy on long-lasting antibody serum titers.

## Methods

### Mice

All animal experiments were conducted on wild-type, B cell-conditional TFEB-deficient mice (TFEB^fl/fl^ Mb1-Cre^+/−^) and littermate control mice (TFEB^fl/fl^ Mb1-Cre^−/−^) on a C57BL/6 background (10-12 weeks). The TFEB^fl/fl^ line was created, first described and kindly gifted to us by Dr. Andrea Ballabio ^26^ and was crossed to the Mb1-Cre deleter strain to obtain B cell-specific TFEB KO animals. Mice were kept under specific-pathogen-free conditions in individually ventilated cages in our animal facilities in Göttingen. Three to five mice per cage were kept at 23 °C, with a 12/12 h day-night cycle. Genotypes were confirmed by PCR analysis of the DNA from ear punch biopsies. We neither anticipated sex-specific effects nor did we observe any in the course of our study. We did, therefore, not restrict our study design to the usage of one particular sex. All animal experiments were conducted according to accepted standards of humane animal care and approved by the responsible authorities in the state of Lower Saxony (Niedersächsisches Landesamt für Verbraucherschutz und Lebensmittelsicherheit).

### Cell culture and treatment

Experiments involving human participants were approved by the ethical review committee of the University Medical Center Göttingen and were performed in accordance with relevant guidelines and regulations. Informed consent was obtained from all participants. Primary human B cells were obtained from the blood of healthy donors. Functional assays were performed on negatively selected B cells enriched by leukocyte reduction system chambers using the MACSxpress LRSC Pan B Cell Isolation Kit (Miltenyi Biotech) according to the manufacturer’s instructions. The human Burkitt lymphoma cell line Ramos (DSMZ ACC603), murine BALB/c-derived IIA1.6 B cells (kindly provided by Dr. Jürgen Frey) and the murine lymphoma cell line WEHI-231 (ATCC CRL-1702), derived from a BALB/c×NZB mouse, were cultured and stimulated with F(ab’)_2_ fragments of anti-IgM and anti-IgG (Jackson Immuno Research). Primary human B cells were stimulated with a mixture of goat-anti human IgM, IgG and IgA F(ab’)_2_ (each 10 µg/ml, Cat. 109-006-129, 190-006-097 and 109-006-011, Jackson Immuno Research). For co-stimulation, 10 µg/ml anti-human CD19 (HIB19, Cat. 302202, Biolegend), 10 µg/ml anti-human CD40 (G28.5, Cat. BE0189, BioXCell) and 10 µg/ml anti-mouse CD40 (HM.40.3, Cat.102914, Biolegend) was used, respectively. The following inhibitors (diluted in DMSO) were used: 2.5 µM BAPTA-AM (Abcam), 2 µM BAY 61-3606 (Calbiochem), 10 µM BIO-acetoxime (Sigma-Aldrich), 10 µM CHIR99021 (Tocris), 10 µM cyclosporin A (Calbiochem), 10 µM Gö6983 (Sigma-Aldrich), 10 µM ibrutinib (Calbiochem), 20 nM leptomycin B (Calbiochem), 25 µM LY294002 (Calbiochem), 100 nM okadaic acid (CST), 25 µM PD98059, 50 µM PP2, 250 nM rapamycin (all Calbiochem), 250 nM torin-1 (Tocris) and 1 µM wortmannin (all Calbiochem).

### Subcellular fractionation

To purify nuclear protein fractions, we performed an up-scaled variation of the ‘Lysis Gradient Centrifugation’ (LGC) protocol published by Katholnig et al.^22^. Per sample, 4 × 10^7^ cells were starved for 30 min in 5 ml serum-free RPMI-1640 at 37 °C. Resting or BCR-stimulated cells were suspended in 2.0 ml PBS containing 5 µg/ml crystal violet (Sigma-Aldrich) and added to an iodixanol-based (VISIPAQUE 320, GE Healthcare) density gradient as the topmost layer. The density gradient consisted of the following layers (bottom to top): ‘floating’ (35% iodixanol in PBS), ‘nuclear wash’ (25% iodixanol in PBS), ‘lysis’ (10% iodixanol, 0.5% NP-40 in PBS), ‘cell wash’ (5% iodixanol in PBS) and the ‘cell suspension’ layer. The ‘nuclear wash’ and ‘lysis’ layers were supplemented with 1 mM sodium vanadate and protease inhibitor cocktail (P2714, Sigma-Aldrich). Gradients were centrifuged for 15 min at 1000 × *g* at 4 °C. Cytosolic proteins were collected from the upper part of the ‘lysis’ layer (600 µl). Intact nuclei (white fuzzy coat) were collected from the top of the ‘floating’ layer. Nuclei were lysed in 200 µl high salt nuclear extraction buffer (20 mM HEPES pH 7.5, 420 mM NaCl_2_, 1.5 mM MgCl_2_, 0.2 mM EDTA, 1 mM Na_3_VO_4_). Lysates were sonicated at 50% amplitude for 5 cycles and 5 sec and incubated for 60 min on ice. Nuclear extracts were cleared by centrifugation at 16,000 × *g* for 20 min at 4 °C. Protein concentrations were measured using the Pierce BCA Protein Assay (Thermo Fisher). Samples (30 µg per lane) were subjected to SDS-PAGE and immunoblotting with antibodies against β-actin (1:1000, 8H10D10, Cat. 3700, CST), ERK (1:2000, clone #16, Cat. 610124, BD), NFAT1 (1:1000, D43B1, Cat. 5861, CST), NF-κB p65 (1:1000, D14E12, Cat. 8242, CST), lamin B1 (1:1000, A11, Cat. sc-377000, Santa-Cruz), hTFEB (1:1000, D2O7D, Cat. 37785, CST), pTFEB S142 (1:500, Cat. ABE1971, Merck Millipore), mTFEB (1:1000, D4L2P, Cat. 32361, CST), α-tubulin (1:1000, Cat. T6199, Sigma-Aldrich) and SAPK/JNK (1:2000, Cat. 9252, CST).

### ‘Translocatome’ SILAC mass spectrometry

Stable isotope labeling with amino acids in cell culture (SILAC) was performed as described^6^. Briefly, cells were metabolically labeled through culturing in medium containing arginine and lysine with incorporated ‘heavy’ isotopes of carbon and nitrogen (^13^C and ^15^N). To allow proteomic profiling under multiple stimulatory conditions, experiments were carried out in ‘SILAC triplets’, consisting of a 1:1:1 mixture of cells cultured with distinct combinations of ‘light’ (Lys+0, Arg+0), ‘medium’ (Lys+4, Arg+6) and ‘heavy’ (Lys+8, Arg+10) amino acids. Per SILAC label, 1.3 × 10^7^ IIA1.6 cells were rested in serum-free RPMI-1640 for 20 min at 37 °C. Cells were left untreated or stimulated with 10 µg/ml anti-mouse IgG. To control for labeling-intrinsic adverse effects, each approach was controlled using a ‘reverse’ replicate, where SILAC labels were switched. In total experiments were carried out in *n* = 2 biological replicates per condition with *n* = 2 technical replicates (reverse label control) per biological replicate. Cells were pooled 1:1:1 to generate ‘SILAC triplets’, containing 3.9 × 10^7^ cells in 2.0 ml PBS and subjected to iodixanol gradient centrifugation-based subcellular fractionation as described above. Mass spectrometric analyzes were performed by the Core Facility Proteomics at the University Medical Center Göttingen.

Samples were reconstituted in 1 × NuPAGE LDS Sample Buffer (Invitrogen) and separated on 4–12% NuPAGE Novex Bis-Tris Minigels (Invitrogen) using half of the gel length. Gels were stained with Coomassie Blue for visualization purposes, and each lane sliced into 11 equidistant regardless of staining. After washing, gel slices were reduced with dithiothreitol (DTT), alkylated with 2-iodoacetamide and digested with Endopeptidase Trypsin (sequencing grade, Promega) overnight. The resulting peptide mixtures were then extracted, dried in a SpeedVac and reconstituted in 2% acetonitrile/0.1% formic acid/ (v:v).

For nanoLC-MS/MS analysis, samples were enriched on a self-packed reversed phase-C18 precolumn (0.15 mm ID × 20 mm, Reprosil-Pur120 C18-AQ 5 µm, Dr. Maisch, Ammerbuch-Entringen, Germany) and separated on an analytical reversed phase-C18 column (0.075 mm ID × 200 mm, Reprosil-Pur 120 C18-AQ, 3 µm, Dr. Maisch) using a 30 min linear gradient of 5–35% acetonitrile/0.1% formic acid (v:v) at 300 nl min-1). The eluent was analyzed on a Q Exactive hybrid quadrupole/orbitrap mass spectrometer (ThermoFisher) equipped with a FlexIon nanoSpray source and operated under Excalibur 2.5 software using a data-dependent acquisition method. Each experimental cycle was of the following form: one full MS scan across the 350–1600 m/z range was acquired at a resolution setting of 70,000 FWHM, and AGC target of 1 × 10e6 and a maximum fill time of 60 ms. Up to the 12 most abundant peptide precursors of charge states 2–5 above a 2 × 10e4 intensity threshold were then sequentially isolated at 2.0 FWHM isolation width, fragmented with nitrogen at a normalized collision energy setting of 25%, and the resulting product ion spectra recorded at a resolution setting of 17,500 FWHM, and AGC target of 2 × 10e5 and a maximum fill time of 60 ms. Selected precursor m/z values were then excluded for the following 15 s. Two technical replicates per sample were acquired.

Raw data were processed using MaxQuant Software version 1.5.2.8 (Max Planck Institute for Biochemistry, Martinsried, Germany). Proteins were identified against the UniProtKB *Mus musculus* reference proteome (v2018.01, 1748 protein entries) along with a set of common lab contaminants. The search was performed with trypsin as an enzyme and iodoacetamide as a cysteine-blocking agent. Up to two missed tryptic cleavages and methionine oxidation as a variable modification were allowed for. The instrument type ‘Orbitrap’ was selected to adjust for MS acquisition specifics. The Arginine R6/R10 and Lysine K4/K8 labels including the ‘Re-quantify’ option were specified for relative protein quantitation by triplex SILAC. Perseus Software version 1.5.0.15 (Max Planck Institute for Biochemistry, Martinsried, Germany) was used to obtain relative protein quantitation values from the MaxQuant Software results and perform statistical evaluation. Nuclear enrichment was assessed by computation of normalized log_2_ SILAC ratios. Proteins with *p* < 0.05 according to significance B analysis were considered to be significantly enriched. The mass spectrometry data generated for this study have been deposited to the ProteomeXchange consortium via the PRIDE partner repository with the dataset identifier PXD054505.

### Imaging flow cytometry

Subsets of primary human B cells were gated based on antibody staining against multiple surface markers: CD27-PE (1:5, M-T271, Cat. 555441, BD), CD19-PE/Cy7 (1:200, HIB19, Cat. 302216, BD), IgD-BV421 (1:20, IA6-2, Cat. 562518, BD) and CD38-APC (1:5, HIT2, Cat. 555462, BD). For subtyping of mouse splenocytes, CD19-APC/Fire750 (1:100, 6D5, Cat. 115558, Biolegend), CD80-BV421 (1:250, 19-10A1, Cat. 104726, Biolegend), CD95(Fas)-Pe/Cy7 (1:100, Jo2, Cat. 562633, BD) and GL7-BV421 (1:250, GL7, Cat. 144620, Biolegend) were used. For intracellular staining, cells were fixed with CytoFix buffer (BD) for 20 min at 4 °C, washed with PBS and resuspended in 200 μl 0.1% TritonX100, 2% FCS in PBS, containing the primary antibody against human TFEB (1:150, D2O7D, Cat. 37785, CST) or murine TFEB (1:250, D4L2P, Cat. 32361, CST). For comparison of TFEB translocation with other transcription factors, antibodies against NFAT1 (1:100, D43B1, Cat. 5861, CST), NF-κB p50 (1:100, E10, Cat. sc-8414, Santa Cruz) and NF-κB p65 (1:100, D14E12, Cat. 8242, CST) were used, respectively. After incubation at 4 °C for 30 min and washing with PBS, cells were stained with a FITC-conjugated secondary anti-rabbit antibody (1:250, #554020, BD) and incubated at 4 °C for 30 min. After washing with PBS, nuclei were stained with 50 µl 10 µg/ml 7-AAD (Thermo Fisher) in PBS. Per sample, at least 10^4^ single, focused cells (as determined via Area vs Aspect Ratio and GradientRMS, respectively) were recorded at the ImageStreamX MkII imaging flow cytometer (Luminex). For subsets of primary human B cells, 10^5^ cells were acquired. Fluorophore compensation and statistical analysis was performed using the IDEAS 6.2 software. Images were acquired in 60x magnification in the following channels: Ch1: Brightfield, Ch2: FITC, Ch3: PE, Ch5: 7-AAD, Ch6: PE/Cy7, Ch7 BV421, Ch9: Brightfield, Ch11: APC, Ch12: SSC or APC/Fire750. Apoptotic cells were excluded by means of their nuclear morphology (‘Apoptosis Wizard’ algorithm). Nuclear localization of TFEB was assessed using the ‘Nuclear Translocation Wizard’ algorithm on the channels 2 (TFEB/FITC) and 5 (Nucleus/7-AAD). For further analyzes, the nuclear area was defined via the mask ‘Dilate(Object(M05,Ch05,Tight),1)’. Events with a ‘SimilarityDilate’ (co-localization score) >1.0, or >1.25 for mouse splenocytes, respectively, were considered cells with nuclear-localized TFEB.

### Flow Cytometry

Cell surface expression of target proteins was analyzed by conventional flow cytometry using the following antibodies. CCR7-BV605 (1:100, 2-L1-A, Cat. 563711, BD), CXCR4-BV421 (1:250, 2B11/CXCR4, Cat. 562738, BD), IL-7R-BV421 (1:100, HIL-7R-M21, Cat. 562436, BD), MHC II (HLA-DR/DP/DQ)-BV421 (1:100, Tu39, Cat. 564224, BD), MHC II (I-A/I-E)-BV421 (1:100, M5/114.15.2, Cat. 562564, BD). Per sample, 1 × 10^6^ Ramos or WEHI-231 cells were left untreated or were stimulated with 10 µg/ml anti-human or anti-mouse IgM F(ab’)_2_ for 6 h and 18 h, as indicated. Staining was performed at 4 °C for 30 min in the dark. For analysis of murine B cell subsets, splenocytes were treated with Zombie Aqua or Zombie Green viability dye (BioLegend), subjected to Fc Blocking (Mouse Fc Block, BD) and surface stained using the following antibodies in Brilliant Buffer (BD): B220-BV605 (1:100, RA3-6BA, Cat. 103244, Biolegend), CD19-APC/Fire750 (1:100, 6D5, Cat. 115558, Biolegend), CD80-BV421 (1:100, 16-10A1, Cat. 104726, Biolegend), CD86-PE (1:500, GL-1, Cat. 105008, Biolegend), CD95(Fas)-BV421 (1:100, Jo2, Cat. 562633, BD), CD95(Fas)-BV786 (1:100, Jo2, Cat. 740906, BD), CXCR4-BV421 (1:250, 2B11/CXCR4, Cat. 562738, BD), GL7-PerCp/Cy5.5 (1:250, GL7, Cat. 144610, BD). Staining was performed at 4 °C for 30 min in the dark. To determine the mitochondrial and lysosomal mass, MitoTracker and LysoTracker reagents (Thermo Fisher) were used, respectively. 1 × 10^6^ Ramos cells were stimulated for 6 h and 24 h with 10 μg/ml anti-human IgM F(ab’)_2_. For experiments with WEHI-231 cells, 5 × 10^5^ cells were stimulated for 24 h and 48 h using 10 μg/ml anti-mouse IgM F(ab’)_2_. Following stimulation, cells were washed and suspended in RPMI-1640 containing 50 nM MitoTracker Green and 50 nM LysoTracker DeepRed. Staining was carried out under mild agitation for 30 min at 37 °C. Cells were washed with PBS and fluorescence was measured via flow cytometry. For intracellular staining of Bcl-xL, 1 × 10^6^ WEHI-231 cells were left untreated or stimulated with anti-mouse IgM F(ab’)_2_ or anti-mouse CD40. Cells were fixed by adding an equal volume of CytoFix (BD) and incubation for 30 min at 4 °C. Cells were stained with 1:100 rabbit anti-Bcl-xL (54HC, Cat. 2764, CST) in 0.1% TritonX100 in PBS for 30 min at 4 °C. Cells were washed with PBS and incubated with 1:250 anti-rabbit-FITC (Cat. 554020, BD) in PBS for 30 min at 4 °C. Fluorescence was measured via flow cytometry. Intracellular calcium mobilization was monitored as described in ref.^72^. For quantification of BCR-induced apoptosis, 1 × 10^5^ WEHI-231 cells were left untreated were stimulated with anti-Ig and/or anti-CD40 as indicated. Per sample, 1 × 10^6^ cells were stained with Annexin V-BV421 and 7-AAD in Annexin-binding buffer, according to the manufacturer’s instructions (BD). Annexin V^−^/7-AAD^−^ double negative cells were considered viable cells, Annexin V^+^/7-AAD^−^ cells were considered to be in early apoptosis, and Annexin V^+^/7-AAD^+^ double positive cells to undergo late apoptosis. For flow cytometric analysis of caspase-3 activation, per sample, 10^6^ cells were left untreated or were stimulated with anti-Ig and/or anti-CD40 as indicated. Cells were fixed with CytoFix buffer (BD) and permeabilized/washed with Perm/Wash I buffer (BD) according to the manufacturer’s instructions. Cells were stained 1:100 with an Alexa Fluor 647 anti-active caspase-3 antibody (Cat. 560626, BD), at 4 °C for 45 min. All flow cytometry experiments were conducted on FACSCelesta or LSRII flow cytometers (BD) and analyzed using FlowJo 10.8 Software.

### Plasmids and retroviral gene transfer

The cDNA encoding human TFEB was purchased from Dharmacon. For site-directed mutagenesis, the cDNA was cloned into the plasmid pCR2.1 (Invitrogen). The integrity of plasmid constructs was confirmed via Sanger sequencing (SeqLab-Microsynth, Göttingen, Germany). For stable expression, all TFEB mutants were N-terminally fused to citrine and cloned into the pMSCVpuro vector (Clontech). Retroviral transductions were performed using the packaging cell line Plat-E (Cell Biolabs), which was maintained in RPMI-1640 + 10% FCS. Transduced cells were selected in the presence of 1 µg/ml puromycin, followed by expression analysis via flow cytometry.

### Genome editing

TFEB-deficient B cell lines were generated by CRISPR/Cas9-mediated genome editing with a guide RNA targeting human TFEB at exon 2 (GCCACCATGGCGTCACGCAT), cloned into the pSpCas9(BB)-2A-GFP vector (Addgene #48138), encoding the targeting guide RNA and the Cas9 protein. The resulting plasmids were transiently transfected using the Amaxa human B cell Nucleofector Kit (Lonza) following the manufacturer’s protocol. 24 h after nucleofection, cells were sorted for high GFP expression and subjected to single-cell dilution and screened for absent TFEB. Additionally, TFEB mutants were generated using the Neon Electroporation system (Thermo Fisher) to directly introduce complexes of target-specific crRNA, tracrRNA and Cas9 protein into Ramos and WEHI-231 cells by electroporation. Specific crRNAs and other reagents of the Alt-R Cas9 system were purchased from IDT. To delete TFEB in Ramos cells, a crRNA targeting exon 3 of the human TFEB gene (GAGTACCTGTCCGAGACCTA) was selected using the IDT custom crRNA design tool. To target TFEB expression in murine WEHI-231 cells, crRNAs targeting either exon 3 (CACGTACTGTCCACCTCGGC) or exon 5 (CCTGAACGTGTACAGCGGTG) were designed. Complexes of crRNA, tracrRNA and Cas9 were prepared using Alt-R reagents (IDT), according to the manufacturer’s instructions. Electroporation in Ramos and WEHI-231 cells was carried out using the Neon electroporation system (Thermo Fisher) at 1400 V, 20 ms for 2 pulses (5 × 10^5^ cells in 10 µl). Cells were subjected to single clone dilution.

### RT-qPCR

The abundance of target transcripts was assessed via RT-qPCR. To that end, 1 × 10^6^ Ramos cells were left untreated or were stimulated with 10 µg/ml anti-human and/or 10 µg/ml anti-mouse CD40 for the indicated time periods. Cells were lysed in TRIzol (Invitrogen) and RNA was isolated according to the manufacturer’s instructions. For cDNA synthesis, 200 ng RNA was processed using the iScript gDNA Clear cDNA Synthesis Kit (Bio-Rad). Quantitative PCR was carried out using the PowerSYBR Green PCR Master-Mix (Invitrogen) according to the manufacturer’s protocol; with 20 ng cDNA and 200 nM of the following primers: BCL2L15 (HRK) fwd: AGGTTGGTGAAAACCCTGTG, BCL2L15 (HRK) rev: GCATTGGGGTGTCTGTTTCT; GAPDH fwd: GGTGTGAACCATGAGAAGTATGA, GAPDH rev: GAGTCCTTCCACGATACCAAAG. Quantitative RT-PCR analyzes were performed using the ABI 7500 Instrument (ThermoFisher). The GAPDH gene was used for normalization, relative expression levels of target genes were calculated with the ΔΔCt method.

### RNA sequencing

For RNA sequencing of TFEB-deficient cell lines, 1.5 × 10^6^ cells were seeded in 4.0 ml RPMI-1640 + 10% FCS. Ramos and WEHI-231 B cells were left untreated or were stimulated with 10 µg/ml anti-human IgM or anti-mouse IgM F(ab’)_2_ for 6 h and 18 h, respectively. Cells were pelleted by centrifugation (300 × *g*) at RT for 5 min and subsequently resuspended in 1.0 ml TRIzol reagent (Ambion) and stored at −80 °C. The quality and integrity of RNA were assessed with a fragment analyzer (Advanced Analytical) using a standard sensitivity RNA analysis kit (DNF-471). All samples selected for sequencing exhibited an RNA integrity number over 7. RNAseq libraries were generated using 200 ng total RNA of a non-stranded RNA Seq, massively-parallel mRNA sequencing approach from Illumina (TruSeq RNA Library Preparation Cat. N°RS-122-2001). Libraries were prepared on the automation workstation (Beckman Colter’s Biomek FXP). For accurate quantitation of cDNA libraries, the fluorometric based QuantiFluor dsDNA system (Promega) was used. The size of the final cDNA libraries was determined using the dsDNA 905 reagent kit (Fragment Analyzer), with a size of 300 bp on average. Libraries were pooled and sequenced on the Illumina HiSeq 4000 (SE; 1 × 50 bp; 30–35 Mio reads/sample). Sequence images were transformed with the Illumina software BaseCaller into BCL files, which were demultiplexed to fastq files with bcl2fastq v2.17.1.14. The quality check was done using FastQC version 0.11.5 (https://www.bioinformatics.babraham.ac.uk/projects/fastqc/, accessed on 17 February 2022). RNA seq data was made public under the GEO accession numbers GSE212456 and GSE212457.

For RNA-sequencing of B and GC B cells derived from the spleens of control or TFEB-KO mice, cells were FACS-sorted and taken up in TRIzol reagent and stored at -80 °C. Total RNA was extracted with the RNA-extraction protocol (Invitrogen). RNA-seq libraries were prepared using the NEBNext Ultra RNA Library Prep Kit (E7530) with minor modifications in ligation (diluting the adapters 1:20) and performing 16 PCR cycles^73^ The libraries were validated as described above and sequenced on an Illumina NovaSeq 6000 with 100 cycles (PE 2x50bp). Libraries were pooled and sequenced on an Illumina NovaSeq 6000 to generate 50 bp paired-end reads. Quality of the sequencing reads was checked using FastQC (v0.12.0) and summarized using MultiQC(v1.14). No data was discarded or trimmed. RNA seq data of TFEB KO mouse samples were made public under the GEO accession number GSE237799.

### Mapping, normalization and gene ontology analysis

Sequences were aligned to the genome reference mus musculus GRCm38 version 100 and homo sapiens GRCh 38 version 100 respectively, using the STAR aligner. Subsequently, read counting was performed using featureCounts. Read counts were analyzed in the R/Bioconductor environment (version 3.4.2) using the DESeq2 package version 1.14.1. Gene annotation was performed using Homo sapiens entries via biomaRt R package version 2.32.1. Differently expressed genes (DEGs) in resting and BCR-stimulated cells were defined by an FDR < 0.01 and a log_2_ fold change of > 1 or < −1. Enrichment of gene ontology terms was computed using ‘GOrilla’ and ‘g:profiler’. Identified regulated biological functions were summarized by removing redundant GO terms and visualized via ‘Revigo’.

For RNA transcriptome analysis of control and TFEB KO mice, the software salmon (1.10.1) was used to generate transcript-level count estimates using selective alignment mode, default options and the Ensembl GRCm39 version 109 as mouse reference genome and transcriptome. For selective alignment, the entire genome was used as a decoy sequence. Furthermore, Kallisto (v0.48.0) was used to map the sequencing reads to the Ensembl GRCm39 reference transcriptome with the option -b 100 to generate 100 bootstrap samples. Estimated transcript-level counts from salmon quantification were imported into R (v4.3), adjusted for transcript length and aggregated to gene-level using tximeta (v1.18.0) Imported counts were normalized and processed for downstream analyzes using DESeq2 (v1.40.2) Differently expressed genes were filtered for an adjusted p-value < 0.05. Estimated transcript-level abundances from kallisto quantification were imported in R using sleuth (v0.30.1) to perform the likelihood-ratio test across genotype while controlling for batch and cell type (design formula full model ~ batch + celltype + genotype). Genes with a q-value < 0.05 were considered differently expressed. Heatmaps were generated using the R package ComplexHeatmap (v2.16.0). Enrichment of gene ontology terms was computed using the ‘g:Profiler’ web application.

### Transwell migration assay

To assess CXCL12-directed migration, 5 × 10^5^ WEHI-231 cells in 75 µl RPMI-1640 + 10% FCS were added to the upper chamber of a 96-well 5 µl pore size transwell plate (Corning). The bottom chambers contained either 235 µl RPMI-1640 + 10% FCS supplemented with 300 ng/ml recombinant mouse CXCL12 (R&D) or a chemokine-free medium to control for unspecific migration. As input control, cell suspension was directly added to the bottom chamber. Cells were allowed to migrate for 4 h at 37 °C, 5% CO_2_. Relative cell numbers were determined using a FACSCelesta flow cytometer (BD) by counting events for 2 min on the ‘high’ flow setting. Specific migration towards CXCL12 (as % of input) was computed from the mean number of cells in the respective bottom chamber using the following formula: $\frac{{{{\rm{mean}}}}\left[{{{\rm{CXCL}}}}12\right]-{{{\rm{mean}}}}\left[{{{\rm{w}}}}/{{{\rm{o\; chemokine}}}}\right]}{{{{\rm{mean}}}}[{{{\rm{input}}}}]}\times 100.$

### Clonogenic survival assay

For clonogenicity analysis of stimulated B cells, wild-type and TFEB KO WEHI-231 cells were left untreated or stimulated with 10 µg/ml anti-mouse IgM F(ab’)_2_ fragments. Upon 48 h of stimulation, cells were diluted and subsequently seeded into 96-well plates to achieve single cell dilutions. Per experiment and condition, 192 wells were analyzed for colony formation 21 days post seeding.

### BH3 profiling

B cells were seeded at 2.5 × 10^5^ cells/ml for 48 h in 384-well plates coated in quadruplicates per condition with 15 µl of 1:50 diluted BH3-peptides in MEB2 buffer (150 mM mannitol, 150 mM KCl, 10 mM HEPES-KOH, 5 mM succinate, 1 mM EGTA, 1 mM EDTA, 0.1% BSA, pH 7.5) containing 0.002% digitonin (Sigma Aldrich), including the controls DMSO (1%) and alamethicin (25 µM, Enzo Life Sciences). Concentration of the used peptides (JPT Peptide Technologies) were as follows: BIM 10/1/0.1 µM, BAD 10/1 µM, HRK 10/1 µM, MS1 10/1 µM, FS1 10 µM, PUMA 10 µM. DMSO was used as vehicle control for full retention of cytochrome c, while alamethicin served as control for complete cytochrome c release. The BCR was stimulated for 8 h and untreated cells served as control. Cells were washed and suspended in MEB2 buffer (1 × 10^6^ cells/ml), followed by incubation of 15 µl cell suspension for 1 h at 25 °C with BH3-peptides and controls. Cells were fixed for 10 min at 25 °C by adding 10 µl of 4% formaldehyde, quenched by the addition of 10 µl N2 buffer (1.7 M Tris base, 1.25 M glycin, pH 9.1) for 10 min at 25 °C and stained overnight by adding 10 µl staining solution (10% BSA, 2% Tween20 in PBS), containing 25 µg/ml Hoechst 33342 (Sigma Aldrich) and 1.25 µg/ml AF647 anti-cytochrome c antibody (6H2.B4, Cat. 612310, Biolegend). Cells were analyzed the next day by flow cytometry (BD LSRFortessa X-20). The DMSO control was used to determine cells with retention of cytochrome c (= no apoptosis). The difference upon BCR stimulation was calculated by the difference between stimulated and unstimulated cells (Δ% cytochrome c release).

### Statistics and Reproducibility

Statistical analysis and data visualization were carried out using GraphPad Prism 9.3. Data is presented as mean ± SD of at least three independent experiments, unless indicated otherwise, and statistical parameters are described in the corresponding figure legend. For immunoblot analyzes, representative blots derived of three independent experiments are shown. Data distribution was assumed to be Gaussian, but was not formally tested. Ordinary (non-paired) one-way ANOVA was used for comparison of variance between group means. Ordinary (non-paired) two-way ANOVA was utilized for comparison between groups affected by two independent variables. When analyzing samples stemming from individual mice, matched (RM) ANOVA analysis was conducted. Statistical significances determined via one-way and two-way ANOVA were corrected for multiple testing using Tukey’s and Dunnett’s method, respectively. Statistical significance is indicated by the corresponding *p* value for each comparison within the respective figure. All data presented in this work is provided in a source data file.

### Reporting summary

Further information on research design is available in the Nature Portfolio Reporting Summary linked to this article.

## Supplementary information

Supplementary Information

Peer Review File

Description of Additional Supplementary Files

Supplementary Data 1

Supplementary Data 2

Supplementary Data 3

Supplementary Data 4

Supplementary Data 5

Reporting Summary

## Source data

Source Data

## Data Availability

The mass spectrometry proteomics data generated for this study have been deposited to the ProteomeXchange consortium via the PRIDE partner repository with the dataset identifier PXD054505. RNA sequencing data generated in this study have been deposited in the GEO repository under the accession codes GSE212456, GSE212457 and GSE237799. Source data are provided in this paper.
