# Supplementary material for: TFEB activation hallmarks antigenic experience of B lymphocytes and directs germinal center fate decisions
Source: Nat Commun. 2024 Aug 14;15:6971. doi: 10.1038/s41467-024-51166-3 (PMC11322606; doi:10.1038/s41467-024-51166-3)
Supplement: Supplementary file 1 — Supplementary Information [file 41467_2024_51166_MOESM1_ESM.pdf]

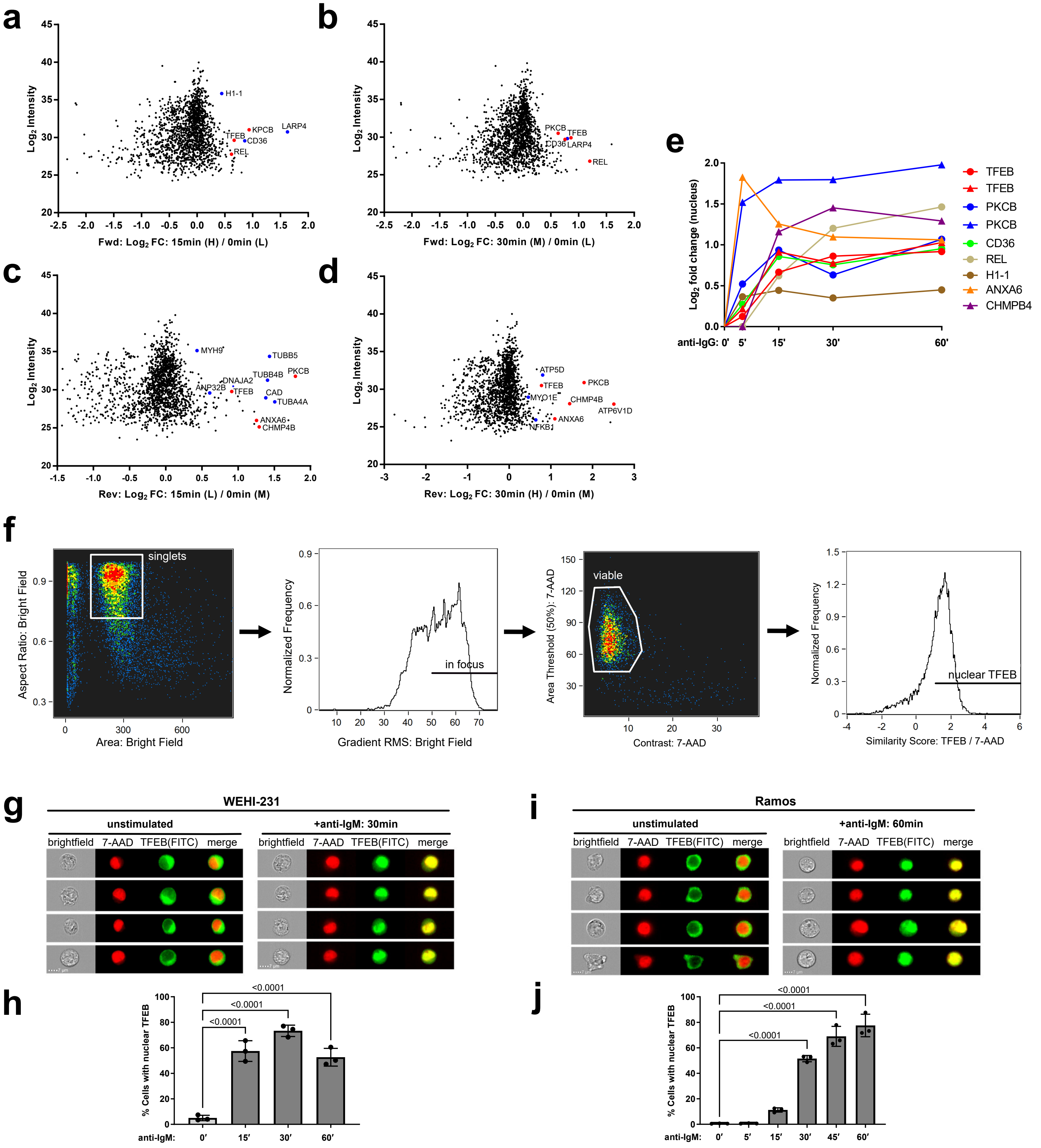
a
b
c
d
e
f
g
h
i
j
Log2 Intensity
Fwd: Log2 FC: 15min (H) / 0min (L)
Fwd: Log2 FC: 30min (M) / 0min (L)
Rev: Log2 FC: 15min (L) / 0min (M)
Rev: Log2 FC: 30min (H) / 0min (M)
H1-1
KPCB
LARP4
TFEB
CD36
REL
PKCB
MYH9
TUBB5
TUBB4B
PKCB
DNAJA2
ANP32B
CAD
TUBA4A
ANXA6
CHMP4B
ATP5D
MYO1E
NFKB1
CHMP4B
ATP6V1D
Log2 fold change (nucleus)
anti-IgG: 0' 5' 15' 30' 60'
TFEB
PKCB
CD36
REL
H1-1
ANXA6
CHMPB4
singlets
Aspect Ratio: Bright Field
Area: Bright Field
Normalized Frequency
in focus
Gradient RMS: Bright Field
viable
Area Threshold (50%): 7-AAD
Contrast: 7-AAD
nuclear TFEB
Similarity Score: TFEB / 7-AAD
WEHI-231
unstimulated
+anti-IgM: 30min
brightfield 7-AAD TFEB(FITC) merge
Ramos
+anti-IgM: 60min
% Cells with nuclear TFEB
anti-IgM: 0′ 15′ 30′ 60′
anti-IgM: 0′ 5′ 15′ 30′ 45′ 60′
<0.0001

Suppl. Fig. 1

Supplementary Fig. 1 | Proteomic replicates of the SILAC-based nuclear translocatome analysis and BCR-induced nuclear translocation of TFEB in B cell lines. (a-d) Scatter plots of identified proteins in the nuclear extracts of the proteomic replicate approach **(a-b)** forward labelling (Fwd) and **(c-d)** reverse labelling (Rev) represented as the $\log_2$-fold enrichment ('SILAC ratios') at the depicted time points, plotted against the $\log_2$ signal intensity. Proteins significantly enriched at two timepoints are highlighted in blue, significantly enriched proteins at ≥3 time points are marked red. **(e)** Nuclear translocation kinetics of significantly enriched proteins in the proteomic replicate approach detected at all time points. Proteins detected in the forward experiment are depicted as circles, proteins identified in the reverse experiment are represented as triangles. **(f)** Gating strategy for the assessment of nuclear translocation of TFEB via imaging flow cytometry: Single cells were gated according to their size (Area) and roundness (Aspect Ratio). Focused cells were defined as having a Gradient RMS value >50. Single cells in focus were further gated according to their double-positive staining of TFEB (FITC) and the nucleus (7-AAD). Apoptotic cells were excluded using the 'apoptosis algorithm' (IDEAS software), analyzing the nuclear staining signal. Nuclear translocation of TFEB in viable cells was finally determined using the 'nuclear translocation algorithm' (IDEAS software) on the channels depicting the TFEB (FITC) and nuclear signal (7-AAD) with a similarity score ≥1. **(g-h)** Resting or BCR-stimulated WEHI-231 B cells (30 min) and **(i-j)** Ramos B cells (60 min) (both n=3) were analyzed for the subcellular distribution of TFEB by imaging flow cytometry as described above and is depicted **(g and i)** as representative images of individual cells and as **(h and j)** mean±SD percentages of translocation-positive cells. Source data are provided as a Source Data file.

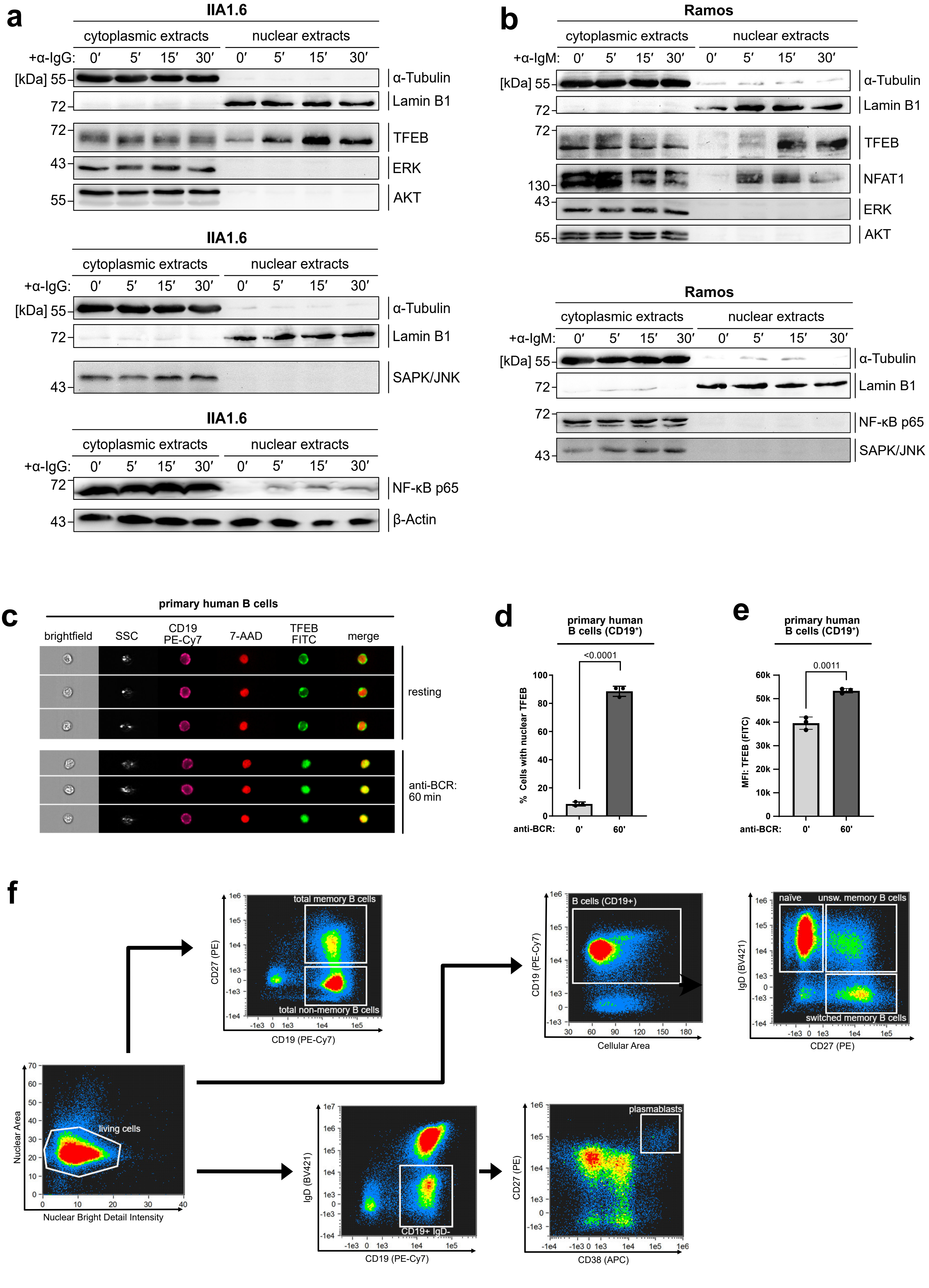
a
IIA1.6
cytoplasmic extracts
nuclear extracts
+α-IgG: 0′ 5′ 15′ 30′ 0′ 5′ 15′ 30′
[kDa] 55
72
72
43
55
α-Tubulin
Lamin B1
TFEB
ERK
AKT
IIA1.6
cytoplasmic extracts
nuclear extracts
+α-IgG: 0′ 5′ 15′ 30′ 0′ 5′ 15′ 30′
[kDa] 55
72
43
α-Tubulin
Lamin B1
SAPK/JNK
IIA1.6
cytoplasmic extracts
nuclear extracts
+α-IgG: 0′ 5′ 15′ 30′ 0′ 5′ 15′ 30′
72
43
NF-κB p65
β-Actin
b
Ramos
cytoplasmic extracts
nuclear extracts
+α-IgM: 0′ 5′ 15′ 30′ 0′ 5′ 15′ 30′
[kDa] 55
72
72
130
43
55
α-Tubulin
Lamin B1
TFEB
NFAT1
ERK
AKT
Ramos
cytoplasmic extracts
nuclear extracts
+α-IgM: 0′ 5′ 15′ 30′ 0′ 5′ 15′ 30′
[kDa] 55
72
72
43
α-Tubulin
Lamin B1
NF-κB p65
SAPK/JNK
c
primary human B cells
brightfield
SSC
CD19 PE-Cy7
7-AAD
TFEB FITC
merge
resting
anti-BCR: 60 min
d
primary human B cells (CD19⁺)
<0.0001
% Cells with nuclear TFEB
0 20 40 60 80 100
anti-BCR: 0' 60'
e
primary human B cells (CD19⁺)
0.0011
MFI: TFEB (FITC)
0 10k 20k 30k 40k 50k 60k
anti-BCR: 0' 60'
f
total memory B cells
total non-memory B cells
CD27 (PE)
CD19 (PE-Cy7)
B cells (CD19+)
CD19 (PE-Cy7)
Cellular Area
naïve
unsw. memory B cells
switched memory B cells
IgD (BV421)
CD27 (PE)
living cells
Nuclear Area
Nuclear Bright Detail Intensity
CD19+ IgD-
IgD (BV421)
CD19 (PE-Cy7)
plasmablasts
CD27 (PE)
CD38 (APC)

Suppl. Fig. 2

Supplementary Fig. 2 | Imaging and biochemical monitoring of the nuclear translocation of various B cell effector proteins. (a-b) Resting or BCR-activated IIA1.6 **(a)** or Ramos **(b)** B cells were subjected to subcellular fractionation via lysis gradient centrifugation. Cytosolic and nuclear fractions were separated by SDS-PAGE and subjected to Western Blot analysis using antibodies recognizing the signaling proteins indicated on the right. Relative molecular masses of marker proteins are indicated on the left in kDa and anti-β-actin staining was used to confirm equal protein loading. **(c-e)** Primary human pan-B cells were isolated from the blood of healthy donors and left untreated or BCR-stimulated for 60 min. TFEB nuclear translocation **(c-d)** and TFEB expression **(e)** of the CD19-positive fraction was analyzed by imaging flow cytometry. Nuclear TFEB was defined by mean similarity scores of TFEB versus 7-AAD. Data are depicted as mean±SD of three independent experiments. **(f)** Multi-color imaging flow cytometric gating strategy of human B cell subsets: Single, focused cells were gated according to their size ('Area') and roundness ('Aspect Ratio'). Upon exclusion of apoptotic and out-of-focus cells (not shown), total non-memory B cells ($CD19^+CD27^-$), total-memory ($CD19^+CD27^+$), naïve ($CD19^+CD27^-IgD^+$), unswitched memory ($CD19^+CD27^+IgD^+$), switched memory ($CD19^+CD27^+IgD^-$) and plasmablasts ($CD19^+CD27^+IgD^-CD38^+$) were distinguished by differential expression of the surface markers CD19, CD27, CD38 and IgD. Source data are provided as a Source Data file.

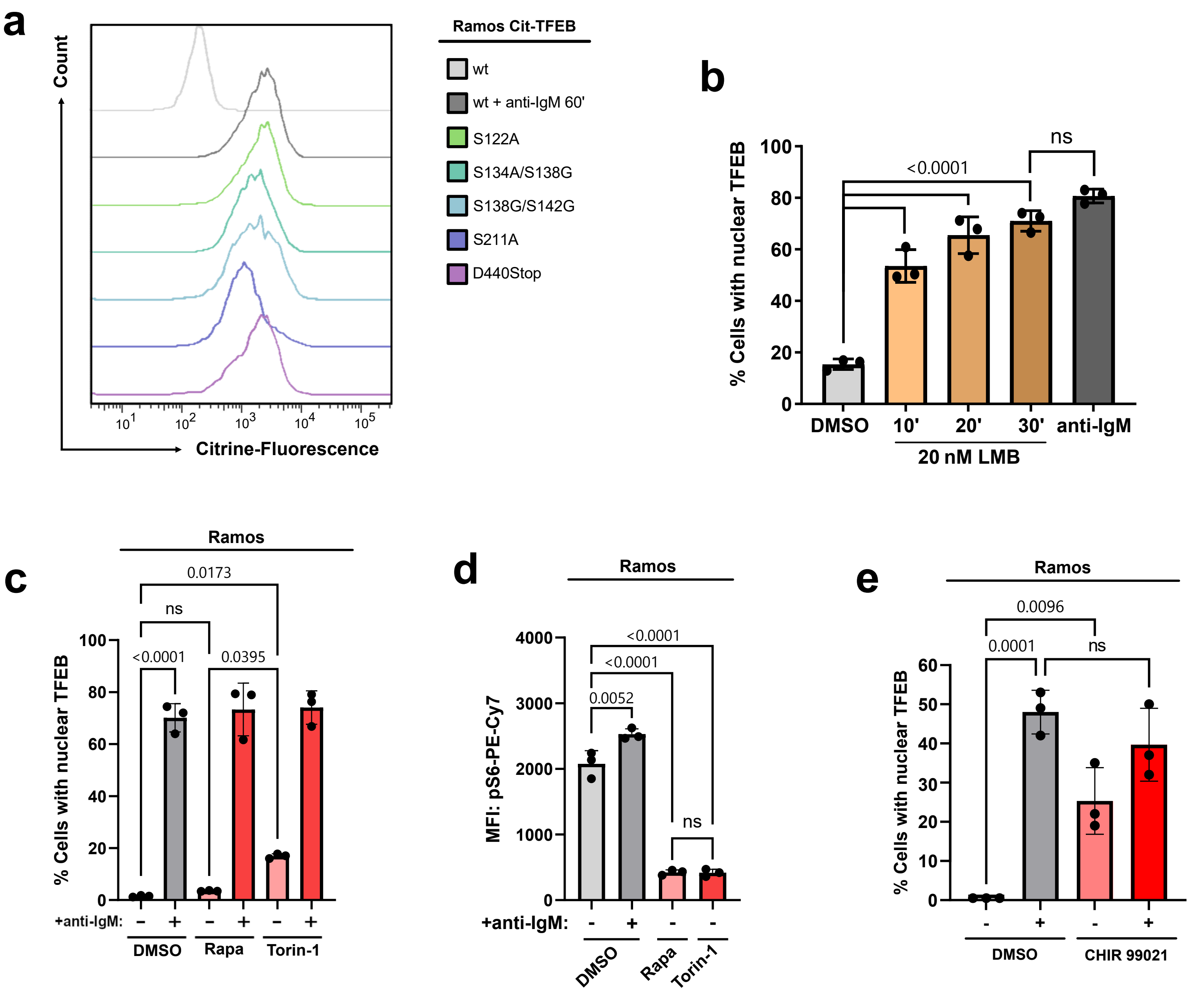
a
Count
Citrine-Fluorescence
Ramos Cit-TFEB
wt
wt + anti-IgM 60'
S122A
S134A/S138G
S138G/S142G
S211A
D440Stop
b
% Cells with nuclear TFEB
<0.0001
ns
DMSO
10'
20'
30'
anti-IgM
20 nM LMB
c
Ramos
% Cells with nuclear TFEB
0.0173
ns
<0.0001
0.0395
+anti-IgM:
DMSO
Rapa
Torin-1
d
Ramos
MFI: pS6-PE-Cy7
<0.0001
<0.0001
0.0052
ns
+anti-IgM:
DMSO
Rapa
Torin-1
e
Ramos
% Cells with nuclear TFEB
0.0096
0.0001
ns
DMSO
CHIR 99021

Suppl. Fig. 3

Supplementary Fig. 3 | Assessment of the regulatory signal network underlying TFEB translocation. (a) Wild-type Ramos cells were retrovirally transduced with citrine-tagged full-length human TFEB or mutated variants of TFEB. Equal expression of the citrine-TFEB fusion protein was confirmed via flow cytometry. **(b)** Ramos B cells were left untreated (-), treated with 20 nM leptomycin B (LMB) for the indicated time periods or BCR-activated and the nuclear translocation of TFEB was monitored by imaging flow cytometry as defined by mean similarity score of TFEB/7-AAD. **(c-e)** Wild-type Ramos B cells were left untreated (-) or BCR-activated (+, 60 min) in the presence of the indicated pharmacological agents: rapamycin (mTOR inhibitor), torin-1 (ATP-competitive mTOR inhibitor) or CHIR 99021 (GSK3 inhibitor). Each of the inhibitors were added to the cells 15 min before BCR ligation and were controlled by treatment of the cells with 1% DMSO. Nuclear TFEB was defined by mean similarity scores of TFEB versus 7-AAD. **(c-d)** Impact of inhibition of mTOR on the nuclear translocation of TFEB as defined by mean similarity scores of TFEB versus 7-AAD **(c)** and on TFEB expression **(d)**. **(e)** Impact of GSK3 inhibition on the nuclear localization of TFEB. Data are depicted as mean±SD of n=3 independent experiments. Statistical significances were computed using one-way **(b)** or two-way **(c-e)** ANOVA and corrected for multiple testing via Tukey's method. Source data are provided as a Source Data file.

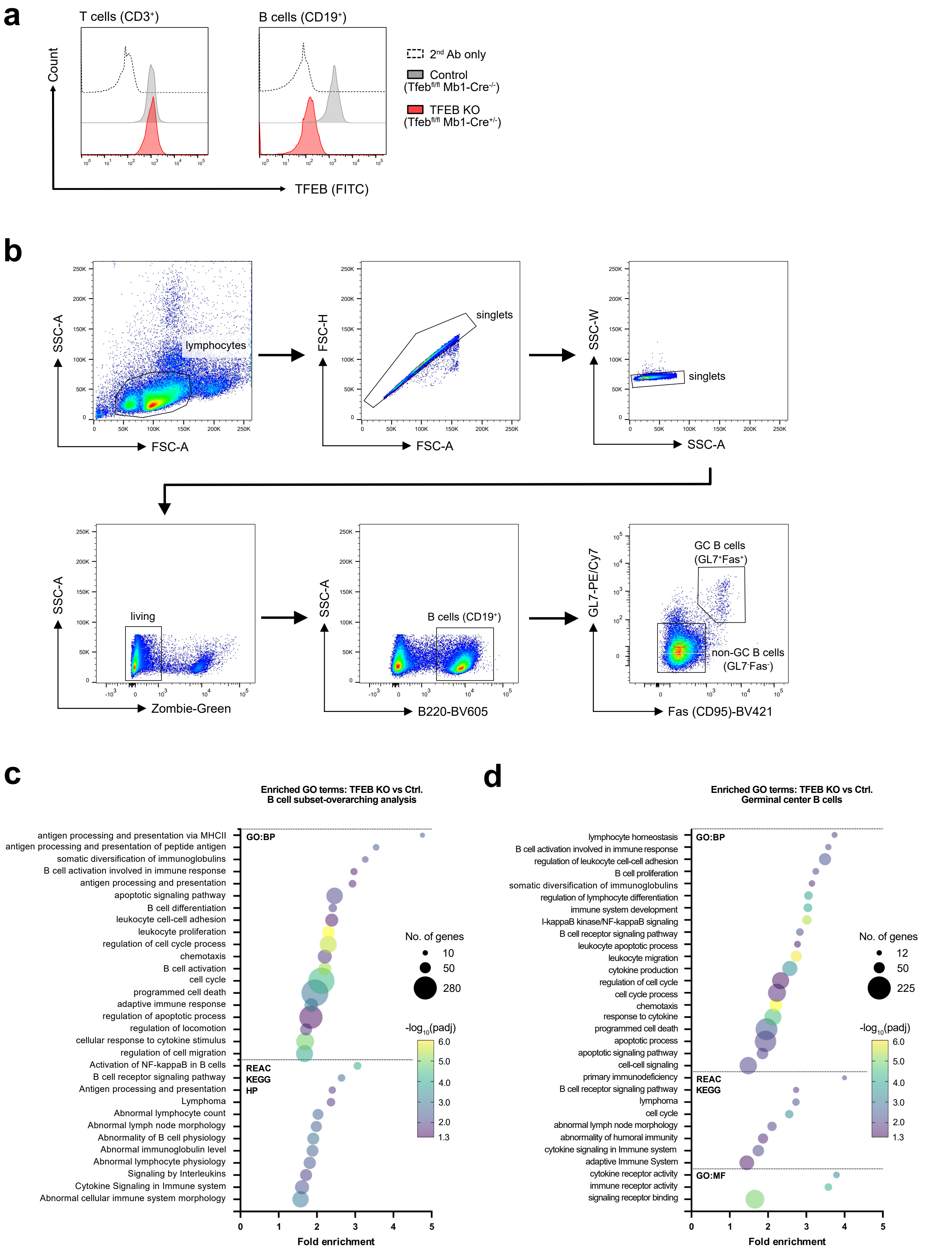

a
T cells (CD3+)
B cells (CD19+)
Count
TFEB (FITC)
2nd Ab only
Control (Tfebfl/fl Mb1-Cre-/-)
TFEB KO (Tfebfl/fl Mb1-Cre+/-)
b
SSC-A
FSC-A
lymphocytes
FSC-H
singlets
SSC-W
SSC-A
singlets
living
Zombie-Green
B cells (CD19+)
B220-BV605
GL7-PE/Cy7
GC B cells (GL7+Fas+)
non-GC B cells (GL7-Fas-)
Fas (CD95)-BV421
c
Enriched GO terms: TFEB KO vs Ctrl.
B cell subset-overarching analysis
GO:BP
antigen processing and presentation via MHCII
antigen processing and presentation of peptide antigen
somatic diversification of immunoglobulins
B cell activation involved in immune response
antigen processing and presentation
apoptotic signaling pathway
B cell differentiation
leukocyte cell-cell adhesion
leukocyte proliferation
regulation of cell cycle process
chemotaxis
B cell activation
cell cycle
programmed cell death
adaptive immune response
regulation of apoptotic process
regulation of locomotion
cellular response to cytokine stimulus
regulation of cell migration
REAC
KEGG
HP
Activation of NF-kappaB in B cells
B cell receptor signaling pathway
Antigen processing and presentation
Lymphoma
Abnormal lymphocyte count
Abnormal lymph node morphology
Abnormality of B cell physiology
Abnormal immunoglobulin level
Abnormal lymphocyte physiology
Signaling by Interleukins
Cytokine Signaling in Immune system
Abnormal cellular immune system morphology
No. of genes
10
50
280
-log10(padj)
6.0
5.0
4.0
3.0
2.0
1.3
Fold enrichment
d
Enriched GO terms: TFEB KO vs Ctrl.
Germinal center B cells
GO:BP
lymphocyte homeostasis
B cell activation involved in immune response
regulation of leukocyte cell-cell adhesion
B cell proliferation
somatic diversification of immunoglobulins
regulation of lymphocyte differentiation
immune system development
I-kappaB kinase/NF-kappaB signaling
B cell receptor signaling pathway
leukocyte apoptotic process
leukocyte migration
cytokine production
regulation of cell cycle
cell cycle process
chemotaxis
response to cytokine
programmed cell death
apoptotic process
apoptotic signaling pathway
cell-cell signaling
REAC
KEGG
primary immunodeficiency
B cell receptor signaling pathway
lymphoma
cell cycle
abnormal lymph node morphology
abnormality of humoral immunity
cytokine signaling in Immune system
adaptive Immune System
GO:MF
cytokine receptor activity
immune receptor activity
signaling receptor binding
No. of genes
12
50
225
-log10(padj)
Fold enrichment

Suppl. Fig. 4

Supplementary Fig. 4 | Gene ontology mapping of TFEB-dependent transcripts in B cells. **(a)** Splenic T cells and B cells from conditional TFEB-deficient mice and control littermates were isolated and analyzed for TFEB expression via flow cytometry. **(b-d)** Splenocytes from age-matched TFEB KO and control littermates were sorted into $CD19^{+}Fas^{+}GL7^{+}$ germinal center (GC) B cells and $CD19^{+}Fas^{-}GL7^{-}$ non-GC B cells and subjected to RNA isolation and bulk RNA sequencing. **(b)** Gating strategy for germinal center and non-GC B cell sorting for RNA sequencing. **(c-d)** Deregulated genes found in the subtype-overarching RNAseq analysis of TFEB-depleted primary B cells, defined as having a qval<0.05, as well as TFEB-dependent DEGs found in murine germinal center B cells, defined by a padj<0.05, were subjected to gene ontology mapping and GO term enrichment analysis using the g:Profiler application. Selected significantly enriched GO terms from the categories "biological process; GO:BP", "molecular function; GO:MF", "biological process; GO:BP", "Reactome database; REAC", "Kyoto Encyclopedia of Genes and Genomes database; KEGG", "human phenotype database; HP" are depicted for the cell type-overarching **(c)** and germinal center B cell analysis **(d)**. The numbers of genes assigned to the respective GO term is illustrated by bubble size, while the $-\log_{10}$ of the adjusted p value is represented through a color gradient. Source data are provided as a Source Data file.

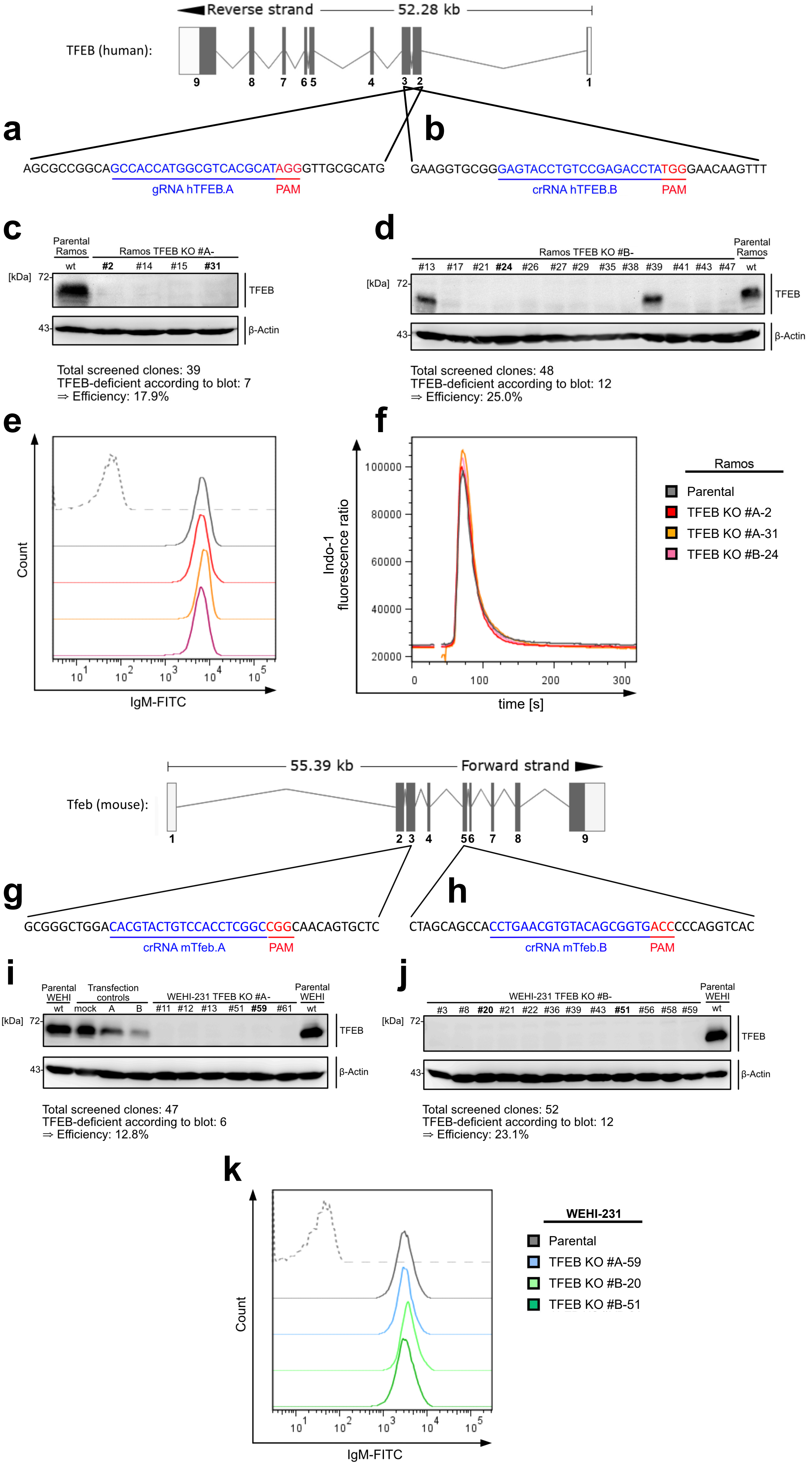
Reverse strand
52.28 kb
TFEB (human):
9 8 7 6 5 4 3 2 1
a
AGCGCCGGCAGCCACCATGGCGTCACGCATAGGGTTGCGCATG
gRNA hTFEB.A
PAM
b
GAAGGTGCGGGAGTACCTGTCCGAGACCTATGGGAACAAGTTT
crRNA hTFEB.B
PAM
c
Parental Ramos
Ramos TFEB KO #A-
wt #2 #14 #15 #31
[kDa] 72-
43-
TFEB
β-Actin
Total screened clones: 39
TFEB-deficient according to blot: 7
⇒ Efficiency: 17.9%
d
Ramos TFEB KO #B-
Parental Ramos
#13 #17 #21 #24 #26 #27 #29 #35 #38 #39 #41 #43 #47 wt
[kDa] 72-
43-
TFEB
β-Actin
Total screened clones: 48
TFEB-deficient according to blot: 12
⇒ Efficiency: 25.0%
e
Count
IgM-FITC
f
Indo-1 fluorescence ratio
100000
80000
60000
40000
20000
0 100 200 300
time [s]
Ramos
Parental
TFEB KO #A-2
TFEB KO #A-31
TFEB KO #B-24
55.39 kb
Forward strand
Tfeb (mouse):
1 2 3 4 5 6 7 8 9
g
GCGGGCTGGACACGTACTGTCCACCTCGGCCGGCAACAGTGCTC
crRNA mTfeb.A
PAM
h
CTAGCAGCCACCTGAACGTGTACAGCGGTGACCCCCAGGTCAC
crRNA mTfeb.B
PAM
i
Parental WEHI
Transfection controls
WEHI-231 TFEB KO #A-
Parental WEHI
wt mock A B #11 #12 #13 #51 #59 #61 wt
[kDa] 72-
43-
TFEB
β-Actin
Total screened clones: 47
TFEB-deficient according to blot: 6
⇒ Efficiency: 12.8%
j
WEHI-231 TFEB KO #B-
Parental WEHI
#3 #8 #20 #21 #22 #36 #39 #43 #51 #56 #58 #59 wt
[kDa] 72-
43-
TFEB
β-Actin
Total screened clones: 52
TFEB-deficient according to blot: 12
⇒ Efficiency: 23.1%
k
Count
IgM-FITC
WEHI-231
Parental
TFEB KO #A-59
TFEB KO #B-20
TFEB KO #B-51

Suppl. Fig. 5

Supplementary Fig. 5 | Generation of TFEB-deficient B cell clones by CRISPR/Cas9-mediated genome editing. (a-f) Targeted gene disruption in and functional analyses of human Ramos B cells. **(a-b)** Schematic representation of the human TFEB gene locus indicating the targeted exons and including PAM sequence. The gRNA utilized for nucleofection is depicted on the left, the crRNA designed for electroporation is shown on the right. **(c-d)** Immunoblot verification of candidate mutant clones using anti-TFEB antibodies. Relative molecular masses of marker proteins are indicated on the left in kDa and anti-β-actin staining was used to confirm equal protein loading. **(c)** Clones generated via nucleofection. **(d)** Clones created via the direct electroporation method. Clones used in the study hereafter are marked in bold. **(e)** BCR surface expression of selected TFEB-deficient clones was monitored by flow cytometry using anti-IgM antibodies. **(f)** Selected clones were examined for their BCR-induced Ca^{2+} mobilization capability by flow cytometry using the ratiometric Ca^{2+} indicator Indo-1 AM. After recording of basal Ca^{2+} levels for 30 s, Ramos B cells were BCR-stimulated with 10 µg/ml anti-IgM $F(ab')_2$ fragments and Ca^{2+} influx was monitored for a total of 300 s. **(g-k)** Targeted gene disruption in and functional analyses of murine WEHI-231 B cells. **(g-h)** Schematic representation of the murine *Tfeb* gene locus indicating the targeted exons and sequences (including PAM). Both crRNAs designed for electroporation are shown. **(i-j)** Immunoblot verification of the candidate mutant clones after the first round of screening. **(i)** Clones generated via the crRNA 'mTfeb.A'. **(j)** Clones created using the 'mTfeb.B' crRNA. TFEB-deficient clones used in the study hereafter are marked in bold. **(k)** Flow cytometric analysis of BCR surface expression of selected TFEB-deficient clones. Data in **(f)** and **(k)** are representative of n=3 independent experiments. Source data are provided as a Source Data file.

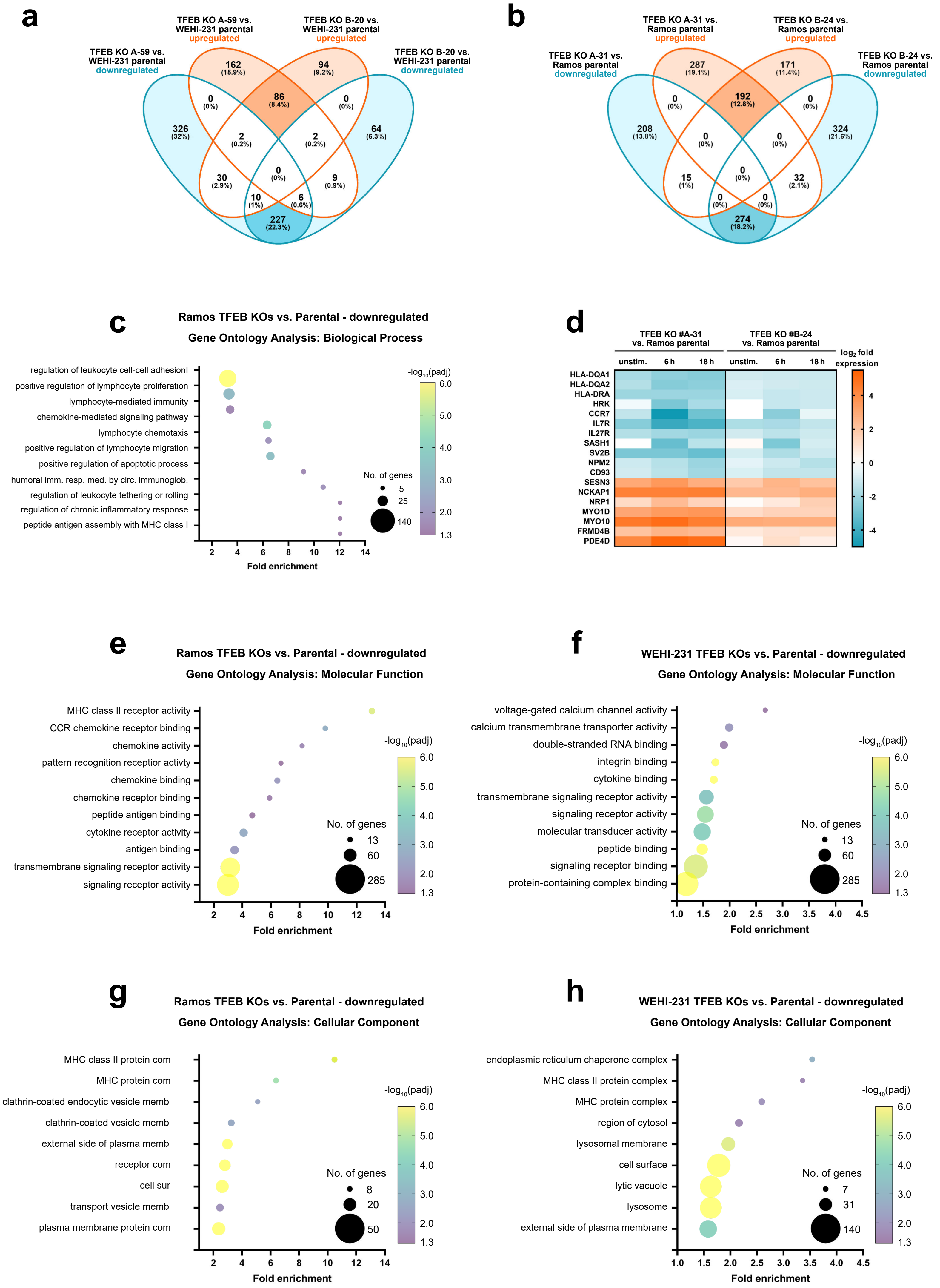
a
TFEB KO A-59 vs. WEHI-231 parental upregulated
TFEB KO B-20 vs. WEHI-231 parental upregulated
TFEB KO A-59 vs. WEHI-231 parental downregulated
TFEB KO B-20 vs. WEHI-231 parental downregulated
162 (15.9%)
94 (9.2%)
0 (0%)
86 (8.4%)
0 (0%)
326 (32%)
2 (0.2%)
2 (0.2%)
64 (6.3%)
30 (2.9%)
0 (0%)
9 (0.9%)
10 (1%)
6 (0.6%)
227 (22.3%)
b
TFEB KO A-31 vs. Ramos parental upregulated
TFEB KO B-24 vs. Ramos parental upregulated
TFEB KO A-31 vs. Ramos parental downregulated
TFEB KO B-24 vs. Ramos parental downregulated
287 (19.1%)
171 (11.4%)
0 (0%)
192 (12.8%)
0 (0%)
208 (13.8%)
0 (0%)
0 (0%)
324 (21.6%)
15 (1%)
0 (0%)
32 (2.1%)
0 (0%)
0 (0%)
274 (18.2%)
c
Ramos TFEB KOs vs. Parental - downregulated
Gene Ontology Analysis: Biological Process
regulation of leukocyte cell-cell adhesionl
positive regulation of lymphocyte proliferation
lymphocyte-mediated immunity
chemokine-mediated signaling pathway
lymphocyte chemotaxis
positive regulation of lymphocyte migration
positive regulation of apoptotic process
humoral imm. resp. med. by circ. immunoglob.
regulation of leukocyte tethering or rolling
regulation of chronic inflammatory response
peptide antigen assembly with MHC class I
Fold enrichment
-log10(padj)
No. of genes
5
25
140
d
TFEB KO #A-31 vs. Ramos parental
TFEB KO #B-24 vs. Ramos parental
unstim.
6 h
18 h
log2 fold expression
HLA-DQA1
HLA-DQA2
HLA-DRA
HRK
CCR7
IL7R
IL27R
SASH1
SV2B
NPM2
CD93
SESN3
NCKAP1
NRP1
MYO1D
MYO10
FRMD4B
PDE4D
e
Ramos TFEB KOs vs. Parental - downregulated
Gene Ontology Analysis: Molecular Function
MHC class II receptor activity
CCR chemokine receptor binding
chemokine activity
pattern recognition receptior activty
chemokine binding
chemokine receptor binding
peptide antigen binding
cytokine receptor activity
antigen binding
transmembrane signaling receptor activity
signaling receptor activity
Fold enrichment
-log10(padj)
No. of genes
13
60
285
f
WEHI-231 TFEB KOs vs. Parental - downregulated
Gene Ontology Analysis: Molecular Function
voltage-gated calcium channel activity
calcium transmembrane transporter activity
double-stranded RNA binding
integrin binding
cytokine binding
transmembrane signaling receptor activity
signaling receptor activity
molecular transducer activity
peptide binding
signaling receptor binding
protein-containing complex binding
Fold enrichment
-log10(padj)
No. of genes
13
60
285
g
Ramos TFEB KOs vs. Parental - downregulated
Gene Ontology Analysis: Cellular Component
MHC class II protein com
MHC protein com
clathrin-coated endocytic vesicle memb
clathrin-coated vesicle memb
external side of plasma memb
receptor com
cell sur
transport vesicle memb
plasma membrane protein com
Fold enrichment
-log10(padj)
No. of genes
8
20
50
h
WEHI-231 TFEB KOs vs. Parental - downregulated
Gene Ontology Analysis: Cellular Component
endoplasmic reticulum chaperone complex
MHC class II protein complex
MHC protein complex
region of cytosol
lysosomal membrane
cell surface
lytic vacuole
lysosome
external side of plasma membrane
Fold enrichment
-log10(padj)
No. of genes
7
31
140

Suppl. Fig. 6

Supplementary Fig. 6 | B-lymphoid TFEB target genes and gene ontology analysis in Ramos and WEHI-231 B cells. Independent TFEB-deficient descendent clones of WEHI-231 **(a-b)** (#A-59 and #B-20) or Ramos (#A-31 and #B-24) **(c-f)** and the respective parental cells were left untreated or BCR-stimulated for 6 h or 18 h, lysed and subjected to RNA sequencing analysis. Data were derived from two independent experiments using two mutant clones per cell line. Numbers of differentially expressed genes (DEGs) defined by an FDR<0.01 and a $\log_2$ fold change of >1 or <-1 are shown as Venn diagrams **(a-b)** depicting the overlap of down- and upregulated DEGs (marked in blue and orange, respectively) in BCR-stimulated WEHI-231 **(a)** and Ramos **(b)** B cells. Downregulated DEGs identified in both of the respective TFEB-depleted WEHI-231 and Ramos cells were subjected to gene ontology analysis. Enrichment of gene ontology terms related to 'biological functions' was computed using 'GOrilla'. Superordinate biological functions were summarized by removing redundant GO terms and visualized via 'Revigo'. The number of genes assigned to the respective GO term is defined by the bubble size, while the adjusted FDR value is represented through a color gradient. **(c)** Selected highly-enriched GO terms reflecting 'biological functions' are depicted for TFEB-deficient versus parental Ramos cells. **(d)** Heatmap depicting $\log_2$ fold changes of selected DEGs with immunological relevance for Ramos mutant clones as compared to the respective parental cells. **(e-f)** Gene ontology enrichment analysis of the molecular functions of identified downregulated DEGs in TFEB-deficient variants of Ramos **(e)** and WEHI-231 **(f)**. **(g-h)** Cellular components associated with identified downregulated DEGs as defined by gene ontology enrichment analysis in TFEB-deficient variants of Ramos **(g)** and WEHI-231 **(h)**. Source data are provided as a Source Data file.

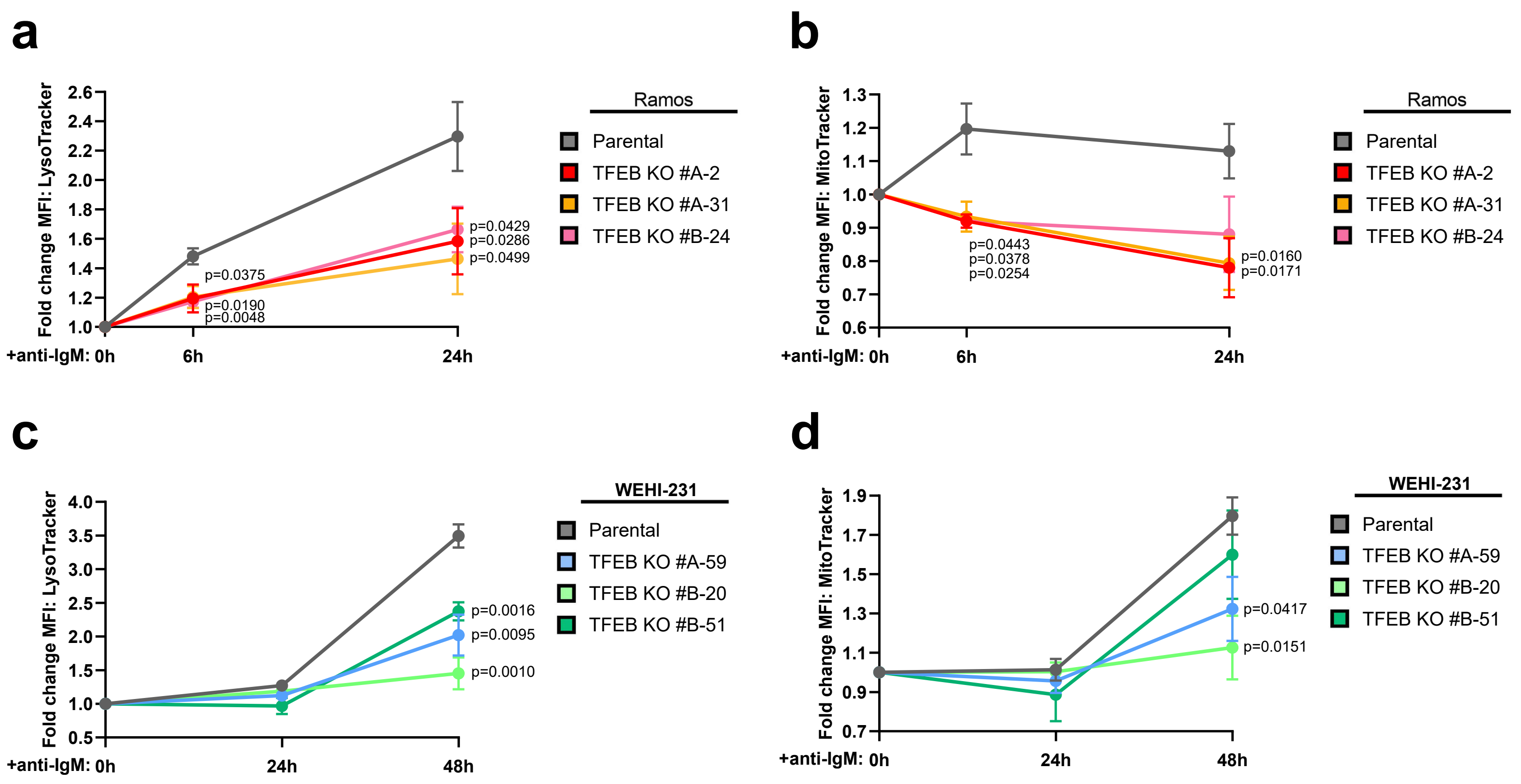
a
Fold change MFI: LysoTracker
2.6
2.4
2.2
2.0
1.8
1.6
1.4
1.2
1.0
+anti-IgM: 0h
6h
24h
p=0.0375
p=0.0190
p=0.0048
p=0.0429
p=0.0286
p=0.0499
Ramos
Parental
TFEB KO #A-2
TFEB KO #A-31
TFEB KO #B-24
b
Fold change MFI: MitoTracker
1.3
1.2
1.1
1.0
0.9
0.8
0.7
0.6
+anti-IgM: 0h
6h
24h
p=0.0443
p=0.0378
p=0.0254
p=0.0160
p=0.0171
Ramos
Parental
TFEB KO #A-2
TFEB KO #A-31
TFEB KO #B-24
c
Fold change MFI: LysoTracker
4.0
3.5
3.0
2.5
2.0
1.5
1.0
0.5
+anti-IgM: 0h
24h
48h
p=0.0016
p=0.0095
p=0.0010
WEHI-231
Parental
TFEB KO #A-59
TFEB KO #B-20
TFEB KO #B-51
d
Fold change MFI: MitoTracker
1.9
1.7
1.5
1.3
1.1
0.9
0.7
+anti-IgM: 0h
24h
48h
p=0.0417
p=0.0151
WEHI-231
Parental
TFEB KO #A-59
TFEB KO #B-20
TFEB KO #B-51

Suppl. Fig. 7

Supplementary Fig. 7 | Lysosomal and mitochondrial adaptation requires TFEB. Ramos **(a-b)** or WEHI-231 B cells **(c-d)** were left untreated or BCR-stimulated for the indicated time periods, and subsequently, lysosomal or mitochondrial masses normalized to the baseline levels were analyzed by flow cytometry using LysoTracker Deep Red **(a and c)** or MitoTracker Green **(b and d)**, respectively. All data are presented as mean±SD of n=3 independent experiments. Statistical significances were calculated using two-way ANOVA and corrected for multiple testing via Dunnett's method. Source data are provided as a Source Data file.

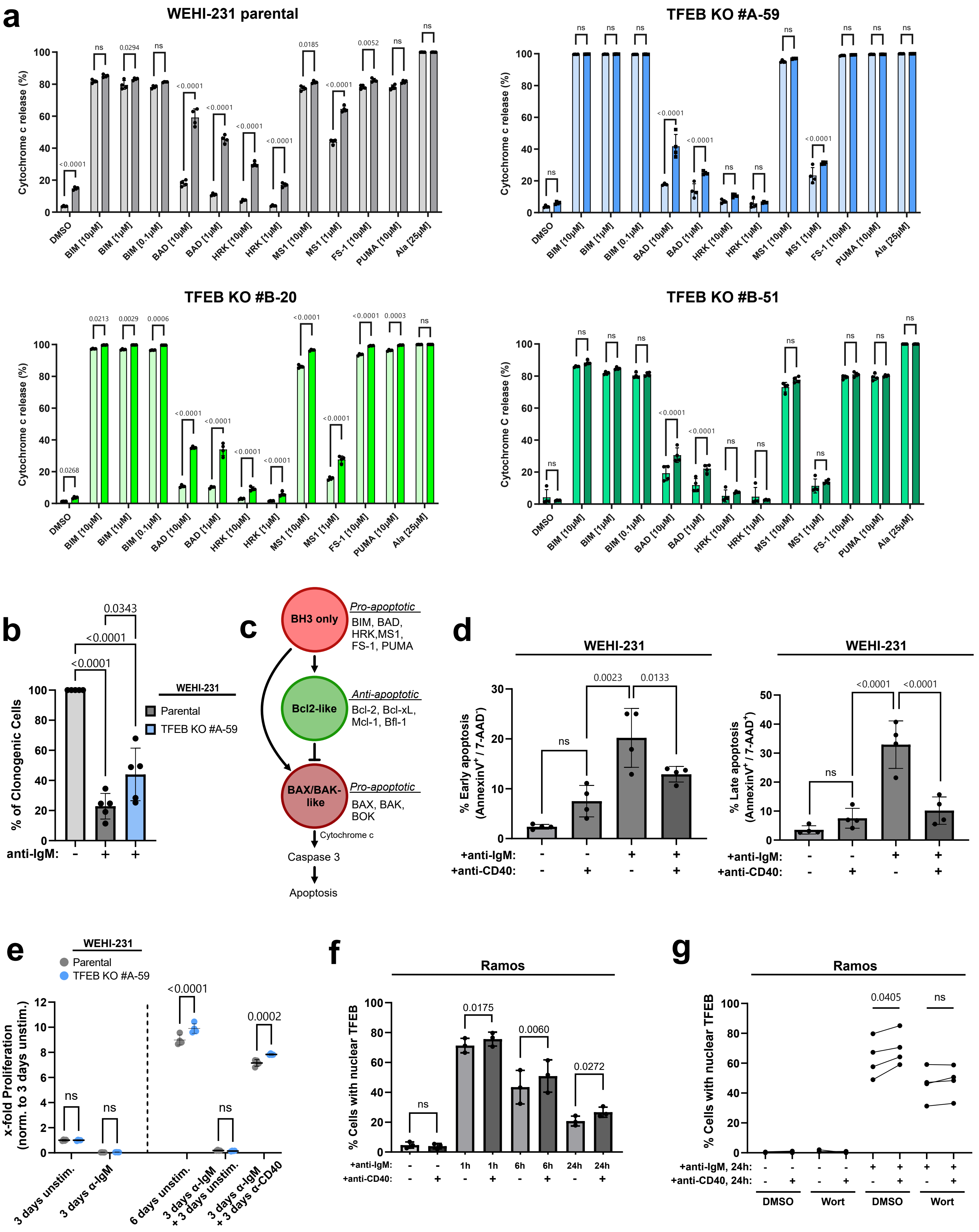
a
WEHI-231 parental
TFEB KO #A-59
TFEB KO #B-20
TFEB KO #B-51
Cytochrome c release (%)
DMSO
BIM [10µM]
BIM [1µM]
BIM [0.1µM]
BAD [10µM]
BAD [1µM]
HRK [10µM]
HRK [1µM]
MS1 [10µM]
MS1 [1µM]
FS-1 [10µM]
PUMA [10µM]
Ala [25µM]
b
% of Clonogenic Cells
WEHI-231
Parental
TFEB KO #A-59
anti-IgM:
c
BH3 only
Pro-apoptotic
BIM, BAD, HRK,MS1, FS-1, PUMA
Bcl2-like
Anti-apoptotic
Bcl-2, Bcl-xL, Mcl-1, Bfl-1
BAX/BAK-like
BAX, BAK, BOK
Cytochrome c
Caspase 3
Apoptosis
d
WEHI-231
% Early apoptosis (AnnexinV+ / 7-AAD-)
% Late apoptosis (AnnexinV+ / 7-AAD+)
+anti-IgM:
+anti-CD40:
e
x-fold Proliferation (norm. to 3 days unstim.)
3 days unstim.
3 days α-IgM
6 days unstim.
3 days α-IgM + 3 days unstim.
3 days α-IgM + 3 days α-CD40
f
Ramos
% Cells with nuclear TFEB
g
+anti-IgM, 24h:
+anti-CD40, 24h:
DMSO
Wort

Supplementary Fig. 8 | Assessment of TFEB-controlled apoptosis and CD40 rescue signals. (a) BH3 profiling of parental cells and three independent TFEB-deficient WEHI-231 cell lines: Percentage of cytochrome c release upon incubation with the indicated relative molecular concentrations of agonistic BH3 peptides was assessed in resting and in BCR-stimulated cells (6 h). The BCR-induced sensitization for apoptosis is presented as the percentage of cytochrome c release of unstimulated (light) and stimulated (dark) cells for each cell line. The presented data is representative of n=3 independent experiments. The depicted data shows the mean%±SD of n=4 technical replicates. Statistical significances were calculated using two-way ANOVA and corrected for multiple testing via Dunnett's method. **(b)** Parental WEHI-231 and TFEB KO #A-59 cells were left untreated or BCR-ligated for 48 h. Single cell dilutions were subsequently seeded in 192 wells per condition and analyzed for clonogenic colony formation for 21 days. **(c)** Schematic depiction of apoptosis regulation through the Bcl-2 protein family pathway: Pro-apoptotic BH3-only proteins activate apoptosis-inducing Bax/Bak-like members through neutralization of anti-apoptotic Bcl-2 family proteins. **(d)** Resting and BCR-activated WEHI-231 B cells were incubated for 48 h in the presence or absence anti-CD40, and cells were monitored by flow cytometry using Annexin V-BV421 and 7-AAD co-staining. **(e)** Parental WEHI-231 and TFEB KO #A-59 cells were left untreated or stimulated with anti-IgM for 3 days. Medium was changed on day 3, and cells were left untreated or were rescued with anti-CD40 until day 6. Cells were harvested on day 3 and day 6 and stained with Annexin V and 7-AAD. The number of viable cells (Annexin V^-/7-AAD^-) was normalized to unstimulated parental cells on day 3. **(f-g)** Ramos B cells were left untreated, or were BCR-ligated for the indicated time periods in the absence or presence of anti-CD40. **(g)** Wortmannin was added 15 min before BCR ligation to antagonize PI3K. Subcellular distribution of TFEB was monitored by imaging flow cytometry using anti-TFEB antibodies and 7-AAD for visualization of the nucleus. Nuclear translocation of TFEB is depicted as percentage of cells with a similarity score of TFEB/7-AAD ≥1 (bottom). Data is presented as mean±SD of n=3 **(f-g)** or n=4 **(b, d and e)** independent experiments. Statistical significances were calculated using one-way ANOVA and corrected for multiple testing via Tukey's method. Source data are provided as a Source Data file.

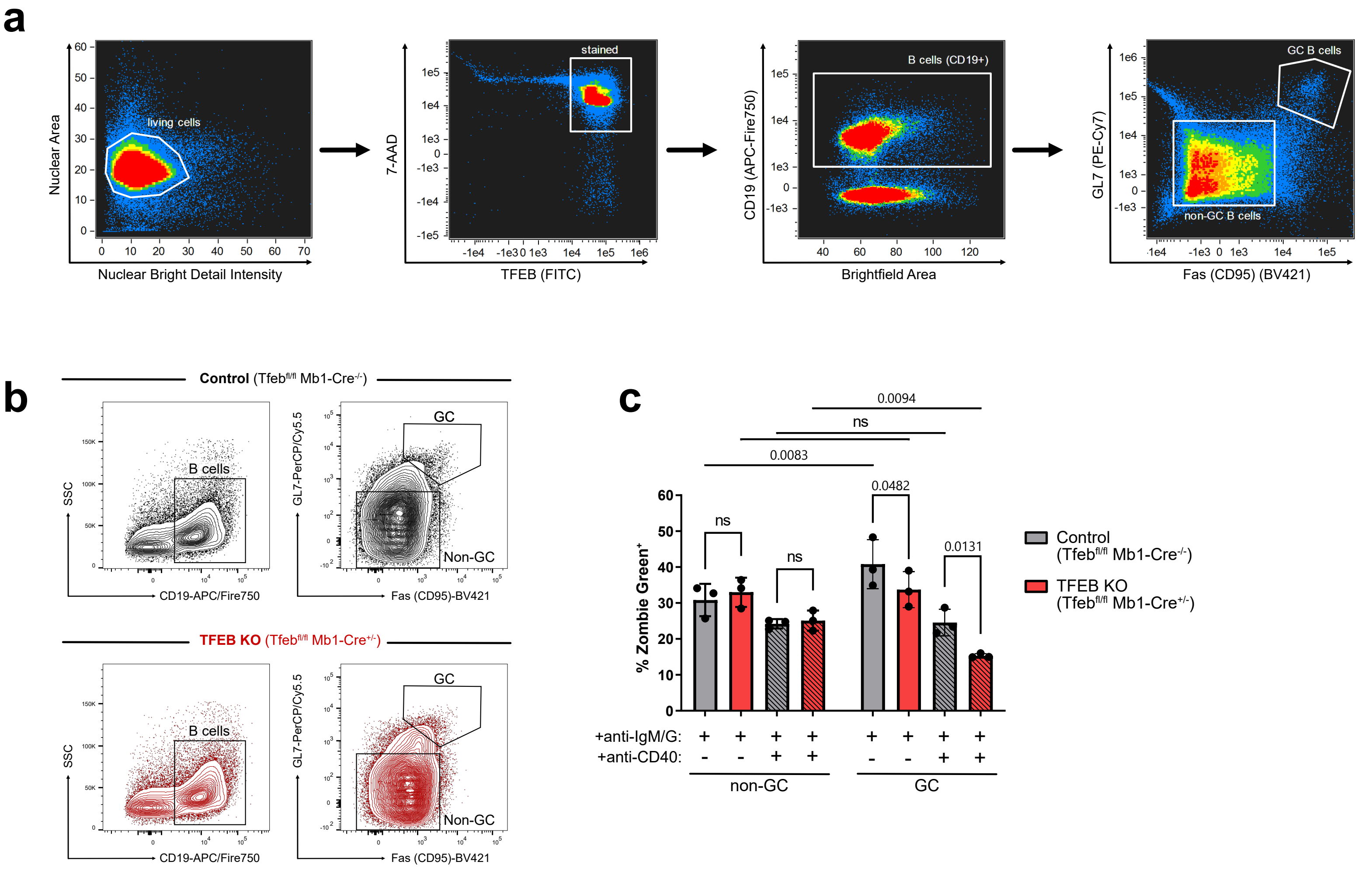
a
living cells
Nuclear Area
Nuclear Bright Detail Intensity
stained
7-AAD
TFEB (FITC)
B cells (CD19+)
CD19 (APC-Fire750)
Brightfield Area
GC B cells
non-GC B cells
GL7 (PE-Cy7)
Fas (CD95) (BV421)
b
Control (Tfeb fl/fl Mb1-Cre-/-)
B cells
SSC
CD19-APC/Fire750
GC
Non-GC
GL7-PerCP/Cy5.5
Fas (CD95)-BV421
TFEB KO (Tfeb fl/fl Mb1-Cre+/-)
c
0.0094
ns
0.0083
0.0482
ns
ns
0.0131
% Zombie Green+
Control (Tfeb fl/fl Mb1-Cre-/-)
TFEB KO (Tfeb fl/fl Mb1-Cre+/-)
+anti-IgM/G:
+anti-CD40:
non-GC
GC

Suppl. Fig. 9

Supplementary Fig. 9 | Analysis of BCR-induced apoptosis in GC B cells. (a) Multi-color imaging flow cytometric gating strategy of germinal center B cells from mouse splenocytes: Upon exclusion of apoptotic and out-of-focus cells (via 'Nuclear Area' and 'Nuclear Bright Detail Intensity') from focused singlets, total B cells were gated as $CD19^{+}$. Total B cells were distinguished by differential expression of the surface markers Fas (CD95) and GL7, with germinal center B cells being defined as $CD19^{+}Fas^{+}GL7^{+}$ and non-GC B cells as $CD19^{+}Fas^{-}GL7^{-}$. **(b-c)** Splenocytes of TFEB-depleted and control mice were isolated and stimulated with anti-IgM/G and co-stimulated with CD40, as indicated, and stained for active caspase-3 and Zombie Green fluorescence. **(b)** Flow cytometric gating strategy: Singlet B cells were distinguished by expression of $CD19^{+}$. Germinal center B cells were gated as $CD19^{+}Fas^{+}GL7^{+}$, whereas non-GC B cells were defined as $CD19^{+}Fas^{-}GL7^{-}$. **(c)** GC and non-GC B cells were gated for high Zombie Green fluorescence. Percentage of Zombie $Green^{+}$ B cells of parental GC or non-GC B cells, respectively, are depicted as mean±SD of n=3 independent experiments. Statistical significances were computed using RM two-way ANOVA and Fisher's LSD test. Source data are provided as a Source Data file.
