# Supplementary material for: TFEB activation hallmarks antigenic experience of B lymphocytes and directs germinal center fate decisions
Source: Nat Commun. 2024 Aug 14;15:6971. doi: 10.1038/s41467-024-51166-3 (PMC11322606; doi:10.1038/s41467-024-51166-3)
Supplement: Supplementary file 3 — Description of Additional Supplementary Files [file 41467_2024_51166_MOESM3_ESM.pdf]

Description of Additional Supplementary Files

Supplementary Data 1 | SILAC MS analysis of the BCR-inducible "Translocatome". IIA1.6 B cells were metabolically labelled via SILAC and either left untreated or BCR-stimulated for 5, 15, 30 or 60 min. Pooled cells were fractionated by iodixanol-based lysis gradient centrifugation. Nuclei were lysed and nuclear proteins were quantified by LC/LC tandem mass spectrometry. For two experiments and separately for the "forward" and "reverse" labeling replicates, the respective table contains $\log_2$ SILAC ratios of all detected proteins, depicting the BCR-induced enrichment of the protein for each timepoint. Statistically significant changes ($p<0.05$, significance B test) are marked with a "+" .

Supplementary Data 2 | Subtype-overarching RNA-seq analysis of murine TFEB-deficient B cells. Splenocytes from TFEB-deficient C57BL/6 mice and control littermates were sorted into $B220^+Fas^+GL7^+$ germinal center (GC) and $B220^+Fas^-GL7^-$ non-GC B cells and subjected to bulk RNA sequencing. For each gene, the table lists the respective number of aggregated transcripts, the sum of the mean observations across all filtered transcripts within the gene, the aggregated p-value calculated by Wald tests as well as the Benjamini-Hochberg-corrected adjusted p-value.

Supplementary Data 3 | RNA-seq analysis of murine TFEB-deficient germinal center B cells. Splenocytes from TFEB-deficient C57BL/6 mice and control littermates were sorted into $B220^+Fas^+GL7^+$ germinal center (GC) and $B220^+Fas^-GL7^-$ non-GC B cells and subjected to bulk RNA sequencing. For each gene, the table list the respective BaseMean expression, the $\log_2$ fold change seen in KO vs. Ctrl. animals and the respective adjusted p-value (Benjamini-Hochberg-corrected likelihood-ratio tests).

Supplementary Data 4 | RNA-seq analysis of TFEB-deficient WEHI-231 cells in the absence and presence of antigenic stimulation. Independent TFEB-deficient WEHI-231 clones (#A-59 and #B-20) and WEHI-231 parental cells were left untreated or BCR-stimulated for 6 or 18 h and subjected to RNA sequencing. In a separate tab for each stimulation condition, the table contains – for each transcript – the $\log_2$ fold expression changes between the respective TFEB KO clones and the parental cell line, as well as the respective FDR value for every comparison.

Supplementary Data 5 | RNA-seq analysis of TFEB-deficient Ramos cells in the absence and presence of antigenic stimulation. Independent TFEB-deficient Ramos clones (#A-31 and #B-24) and Ramos parental cells were left untreated or BCR-stimulated for 6 or 18 h and subjected to RNA sequencing. In a separate tab for each stimulation condition, the table contains – for each transcript – the $\log_2$ fold expression changes between the respective TFEB KO clones and the parental cell line, as well as the respective FDR value for every comparison.
