# Supplementary material for: TFEB activation hallmarks antigenic experience of B lymphocytes and directs germinal center fate decisions
Source: Nat Commun. 2024 Aug 14;15:6971. doi: 10.1038/s41467-024-51166-3 (PMC11322606; doi:10.1038/s41467-024-51166-3)
Supplement: Supplementary file 9 — Reporting Summary [file 41467_2024_51166_MOESM9_ESM.pdf]

nature portfolio

Corresponding author(s): Jürgen Wienands

Last updated by author(s): Jun 26, 2024

Reporting Summary

Nature Portfolio wishes to improve the reproducibility of the work that we publish. This form provides structure for consistency and transparency in reporting. For further information on Nature Portfolio policies, see our Editorial Policies and the Editorial Policy Checklist.

Statistics

For all statistical analyses, confirm that the following items are present in the figure legend, table legend, main text, or Methods section.

n/a	Confirmed	
☐	☒	The exact sample size (*n*) for each experimental group/condition, given as a discrete number and unit of measurement
☐	☒	A statement on whether measurements were taken from distinct samples or whether the same sample was measured repeatedly
☐	☒	The statistical test(s) used AND whether they are one- or two-sided<br>*Only common tests should be described solely by name; describe more complex techniques in the Methods section.*
☒	☐	A description of all covariates tested
☐	☒	A description of any assumptions or corrections, such as tests of normality and adjustment for multiple comparisons
☐	☒	A full description of the statistical parameters including central tendency (e.g. means) or other basic estimates (e.g. regression coefficient) AND variation (e.g. standard deviation) or associated estimates of uncertainty (e.g. confidence intervals)
☐	☒	For null hypothesis testing, the test statistic (e.g. *F*, *t*, *r*) with confidence intervals, effect sizes, degrees of freedom and *P* value noted<br>*Give P values as exact values whenever suitable.*
☒	☐	For Bayesian analysis, information on the choice of priors and Markov chain Monte Carlo settings
☒	☐	For hierarchical and complex designs, identification of the appropriate level for tests and full reporting of outcomes
☒	☐	Estimates of effect sizes (e.g. Cohen's *d*, Pearson's *r*), indicating how they were calculated

Our web collection on statistics for biologists contains articles on many of the points above.

Software and code

Policy information about availability of computer code

Data collection	FACSDiva v9.0 (BD Biosciences), ISX (Luminex Corp.), ChemoStar (INTAS)
Data analysis	FlowJo v10.8.1 (Treestar), IDEAS v6.2 (Luminex Corp.), Fiji ImageJ v1.53c, GOrilla (http://cbl-gorilla.cs.technion.ac.il), Revigo (https://revigo.irb.gr), g:Profiler (https://biit.cs.ut.ee/gprofiler), Graphpad Prism v9.4.0, salmon v1.10.1, kallisto v0.48.0, R v4.3, tximeta v1.18.0, sleuth v0.30.1

For manuscripts utilizing custom algorithms or software that are central to the research but not yet described in published literature, software must be made available to editors and reviewers. We strongly encourage code deposition in a community repository (e.g. GitHub). See the Nature Portfolio guidelines for submitting code & software for further information.

Data

Policy information about availability of data

All manuscripts must include a data availability statement. This statement should provide the following information, where applicable:

- Accession codes, unique identifiers, or web links for publicly available datasets
- A description of any restrictions on data availability
- For clinical datasets or third party data, please ensure that the statement adheres to our policy

All data to support and evaluate the findings presented in this study are included in this article's main text or in the provided supplementary information and/or Source data file. RNA sequencing data was made publicly available on the GEO repository under the accession numbers GSE212456, GSE212457 and GSE237799.

Human research participants

Policy information about studies involving human research participants and Sex and Gender in Research.

Reporting on sex and gender	Blood was obtained from anonymous, healthy donors. Biometrical data of donors was not disclosed to us.
Population characteristics	Blood was obtained from anonymous, healthy donors. Biometrical data of donors was not disclosed to us.
Recruitment	Blood was obtained from anonymous, healthy donors. A recruitment for donors with specific characteristics was not conducted.
Ethics oversight	Experiments involving human participants were approved by the ethical review committee of the University Medical Center Göttingen (case number 11/6/17) and were performed in accordance with relevant guidelines and regulations. An informed consent was obtained from the participants.

Note that full information on the approval of the study protocol must also be provided in the manuscript.

Field-specific reporting

Please select the one below that is the best fit for your research. If you are not sure, read the appropriate sections before making your selection.

[x] Life sciences [ ] Behavioural & social sciences [ ] Ecological, evolutionary & environmental sciences

For a reference copy of the document with all sections, see nature.com/documents/nr-reporting-summary-flat.pdf

Life sciences study design

All studies must disclose on these points even when the disclosure is negative.

Sample size	Our manuscipt does not include human or anmial cohort studies. Therefore, a priori determination of sample size was not conducted. The sample sizes of our biochemical and imaging experiments to assess cell biological responses were conducted to sufficiently assess statistical significance.
Data exclusions	Data were excluded only in the case of apparent technical errors such as faulty positive or negative controls.
Replication	All experiments were replicated in at least two (>3, in most cases) independent replicates and findings were confimed with statistical significance. All attempts at replication were successful.
Randomization	Randomization was less relevant due to the nature of our ex vivo/ in vitro methodology, since the same population of cells were split and used for multiple experimental groups (e.g untreated vs. stimulated) in parallel. For ex vivo experimentation, care was taken to minimize litter effects by using littermates. Sample acquisition was carried out in alternating fashion for KO and control samples.
Blinding	For in vitro experiments, Individual investigators were both collecting and analyzing experimental data and could therefore not be blinded. Sample aquisition of mouse cells was carried out blinded, without knowledge of the assigned genotype.

Reporting for specific materials, systems and methods

We require information from authors about some types of materials, experimental systems and methods used in many studies. Here, indicate whether each material, system or method listed is relevant to your study. If you are not sure if a list item applies to your research, read the appropriate section before selecting a response.

Materials & experimental systems

n/a	Involved in the study	
[ ]	[x]	Antibodies
[ ]	[x]	Eukaryotic cell lines
[x]	[ ]	Palaeontology and archaeology
[ ]	[x]	Animals and other organisms
[x]	[ ]	Clinical data
[x]	[ ]	Dual use research of concern

Methods

n/a	Involved in the study	
[x]	[ ]	ChIP-seq
[ ]	[x]	Flow cytometry
[x]	[ ]	MRI-based neuroimaging

Antibodies

Antibodies used

Antibodies used for B cell stimulation:
Anti-human IgM F(ab')2 (10µg/ml) (Cat.109-006-129, Jackson ImmunoResearch)
Anti-human IgG F(ab')2 (10µg/ml) (Cat.190-006-097, Jackson ImmunoResearch)
Anti-human IgA F(ab')2 (10µg/ml) (Cat.109-006-011, Jackson ImmunoResearch)
Anti-human CD19 (5-10µg/ml, as indicated) (HIB19, Cat. 302202, Biolegend)
Anti-human CD40 (10µg/ml) (G28.5, Cat. BE0189, BioXCell)
Anti-mouse IgM F(ab')2 (10µg/ml) (Cat.115-006-075, Jackson ImmunoResearch)
Anti-mouse IgG F(ab')2 (10µg/ml) (Cat.115-006-071, Jackson ImmunoResearch)
Anti-mouse CD40 (10µg/ml) (HM.40.3, Cat.102914, Biolegend)

Antibodies used for imaging flow cytometry:
Anti-human TFEB (1:150) (D2O7D, Cat. 37785, Cell Signaling Technology)
Anti-mouse TFEB (1:250) (D4L2P, Cat. 32361, Cell Signaling Technology)
Anti-mouse NFAT1 (1:100) (D43B1, Cat. 5861, Cell Signaling Technology)
Anti-mouse NF-kB p50 (1:100) (E10, Cat. sc-8414, Santa Cruz)
Anti-mouse NF-kB p65 (1:100) (D14E12, Cat. 8242S, Cell Signaling Technology)
Anti-rabbit IgG-FITC (human: 1:150, mouse: 1:250) (Cat. 554020, BD Biosciences)
Anti-human IgD -BV421 (1:20) (IA6-2, Cat. 562518, BD Biosciences)
Anti-human CD19-PE-Cy7 (1:200) (HIB19, Cat. 302216, BD Biosciences)
Anti-human CD27-PE (1:5) (M-T271, Cat. 555441, BD Biosciences)
Anti-human CD38-APC (1:5) (HIT2, Cat. 555462, BD Biosciences)
Anti-mouse CD19-APC/Fire750 (1:100) (6D5, Cat. 115558, BioLegend)
Anti-mouse GL7-PE/Cy7 (1:250) (GL7, Cat. 144620, BioLegend)
Anti-mouse Fas (CD95)-BV421 (1:100) (Jo2, Cat. 562633, BD)
Anti-mouse CD80-BV421 (1:250) (16-10A1, Cat. 104726, BioLegend)

Antibodies used for immunoblotting:
Anti-beta-Actin (1:1000) (8H10D10, Cat. 3700, Cell Signaling Technology)
Anti-ERK (1:2000) (#16, Cat. 610124, BD Biosciences)
Anti-Lamin B1 (1:1000) (A11, Cat. sc-377000, Santa-Cruz)
Anti-NFAT1 (1:1000) (D43B1, Cat. 5861, Cell Signaling Technology)
Anti-NF-kB p65 (1:1000) (D14E12, Cat. 8242, Cell Signaling Technology)
Anti-SAPK/JNK (1:2000) (9252T, Cat. 9252, Cell Signaling Technology)
Anti-SWP70 (1:1000) (D5O6A, Cat. 33942, Cell Signaling Technology)
Anti-human TFEB (1:1000) (D207D, Cat. 37785, Cell Signaling Technology)
Anti-mouse TFEB (1:1000) (D4L2P, Cat. 32361 Cell Signaling Technology)
Anti-phospho TFEB S142 (1:500) (Cat. ABE1971, Merck-Millipore)
Anti-alpha-Tubulin (1:1000) (Cat. T6199, Sigma-Aldrich)
Anti-mouse IgG1-HRP (1:10000) (Cat.1070-05, Southern Biotech)
Anti-mouse IgG2a-HRP (1:10000) (Cat.1080-05, Southern Biotech)
Anti-mouse IgG2b-HRP (1:10000) (Cat.1090-05, Southern Biotech)
Anti-rabbit-IgG-HRP (1:10000) (Cat.4050-05, Southern Biotech)

Antibodies used for flow cytometry:
Anti-human MHCII (HLA DR/DP/DQ)-BV421 (1:100) (Tu39, Cat. 564224, BD Biosciences)
Anti-human CCR7-BV605 (1:100) (2-L1-A, Cat. 563711, BD Biosciences)
Anti-human IL-7R-BV421 (1:100) (HIL-7R-M21, Cat. 562436, BD Biosciences)
Anti-Bcl-xL (1:100) (54H6, Cat. 2764, Cell Signaling Technology)
Anti-mouse CD184 (1:250) (CXCR4) (2B11/CXCR4, Cat. 562738, BD Biosciences)
Anti-mouse MHCII (I-A/I-E) (1:100) (M5/114.15.2, Cat. 562564, BD Biosciences)
Anti-Cytochrome c-AlexaFluor647 (1.25µg/ml) (6H2.B4, Cat. 612310, Biolegend)
Anti-Active Caspase-3-AlexaFluor647 (1:100) (Cat. 560626, BD)
Anti-mouse B220-BV605 (1:100) (RA3-6BA, Cat. 103244, Biolegend)
Anti-mouse CD19-APC/Fire750 (1:100) (6D5, Cat. 115558, BioLegend)
Anti-mouse GL7-PerCp/Cy5.5 (1:250) (GL7, Cat. 144610, BD)
Anti-mouse CD95 (Fas)-BV421 (1:100) (Jo2, Cat. 562633, BD)
Anti-mouse CD95 (Fas)-BV786 (1:100) (Jo2, Cat. 740906, BD)
Anti-mouse CD80-BV421 (1:250) (16-10A1, Cat. 104726, BioLegend)
Anti-mouse CD86-PE (1:500) (GL-1, Cat. 105008, Biolegend)

Other reagents used for flow cytometry:
7-AAD (10µg/ml) (Cat. A1310, Thermo Fisher)
AnnexinV-BV421 (1:100) (Cat. 563973, BD)
MitoTracker Green (50nM) (Cat. M7514, Thermo Fisher)
LysoTracker Deep Red (50nM) (Cat. M22426, Thermo Fisher)

ZombieAqua (1:500) (Cat. 423102, Biolegend)
ZombieGreen (1:500) (Cat. 423112, Biolegend)

Validation

All listed antibodies were commercially available and validated by the respective manufacturer. For the anti-mouse and anti-human TFEB antibodies, specificity could be validated through TFEB-depleted mice as well as through knockout cell lines of human and murine origin.

Eukaryotic cell lines

Policy information about cell lines and Sex and Gender in Research

Cell line source(s)

IIA1.6 cells were kindly provided by Dr. Jürgen Frey.
Ramos cells were originally from DSMZ (ACC 603).
WEHI-231 cells were originally from ATCC (CRL-1702).

Authentication

Cell lines were authenticated by the respective commercial vendor and used in low passage numbers. Cell lines were authenticated based on morphology and proliferation characteristics as well as via FACS phenotyping of well-defined surface markers.

Mycoplasma contamination

All cell lines tested negative for Mycoplasma contamination as verified via routine PCR testing.

Commonly misidentified lines
(See ICLAC register)

No commonly misidentified cell lines were used.

Animals and other research organisms

Policy information about studies involving animals; ARRIVE guidelines recommended for reporting animal research, and Sex and Gender in Research

Laboratory animals

C57BL/6 TFEB fl/fl Mb1-Cre+/- (KO) and TFEB fl/fl Mb1-Cre-/- (Control) mice were used within this study. All mice were sacrificed at the age of 10-12 weeks. Three to five mice per cage were kept in individually ventilated cages, at 23°C, with a 12h/12h day night cycle.

Wild animals

No wild animals were used.

Reporting on sex

We neither anticipated sex-specific effects, nor did we observe any in the course of our study. We did therefore not restrict our study design to the usage of one particular sex.

Field-collected samples

No field-collected samples were used.

Ethics oversight

All animal experiments were conducted according to accepted standards of humane animal care and approved by the responsible authorities, specifically by the Lower Saxony State Office for Consumer Protection and Food Safety ("Niedersächsisches Landesamt für Verbraucherschutz und Lebensmittelsicherheit").

Note that full information on the approval of the study protocol must also be provided in the manuscript.

Flow Cytometry

Plots

Confirm that:

- [x] The axis labels state the marker and fluorochrome used (e.g. CD4-FITC).
- [x] The axis scales are clearly visible. Include numbers along axes only for bottom left plot of group (a 'group' is an analysis of identical markers).
- [x] All plots are contour plots with outliers or pseudocolor plots.
- [x] A numerical value for number of cells or percentage (with statistics) is provided.

Methodology

Sample preparation

Single cell suspensions of human and murine cell lines were seeded in RPMI+10% FCS, stimulated, washed with PBS and directly used for (imaging) flow cytometry experiments.

Primary human B cells were derived from the blood of healthy donors, collected via the leucocyte reduction chamber system (LRSC). Enrichment of B cells was performed using the MACSxpress LRSC Pan B Cell Isolation Kit (Miltenyi Biotech) according to the manufacturer's instructions. Resulting single cell suspensions were seeded in RPMI+10% FCS, stimulated, washed with PBS and directly used for imaging flow cytometry experiments.

Murine splenocytes and cells from Peyer's patches were homogenized with a 18 G needle and and filtered through a 100 µm cell strainer to obtain a homogeneous cell suspension. Cells were washed in PBS, resuspended in red blood cell lysis (0.16 M

NH4Cl, 0.11 M KHCO3, 1 mM EDTA in H2O) and incubated on for 4 min, then washed with PBS. Resulting single cell suspensions were seeded in RPMI+10% FCS, were stimulated/treated, washed with PBS and directly used for subsequent flow cytometry experiments.

Instrument

Flow Cytometry data was collected on LSRII and FACSCelesta instruments (BD Biosciences).
Imaging flow cytometry data was aquiered on the ImageStreamX MKII (Luminex).
Samples for scRNAseq were sorted on the FACSAria III (BD) instrument with minimal flow speed.

Software

Flow cytometry data was collected using FACSDiva software (BD Biosciences) and analyzed using FlowJo v10.8 (Treestar).
Imaging flow cytometry data was collected using ISX software (Luminex) and analyzed using IDEAS v6.2 (Luminex).

Cell population abundance

Enrichment of primary B cells from LRSCs yielded purities of 85-95%, as determined by anti-CD19 staining.
FACS sorting of primary murine splenocyted yielded >85% germinal center B cells, as defined by (post-sort) GL7+Fas+ positivity.

Gating strategy

Imaging flow cytometry gating strategies for cell lines and primary cell (sub-)populations are depicted in Supplementary Fig. 1f and Supplementary Fig. 2f, Fig. 8a and Supplementary Fig. 9a, respectively. Cells were gated for low "Area of Ch1" ("size"), high “AspectRatio” of Ch1 ("roundness") and high “GradientRMS of Ch1” ("in focus"). Human cells were gated by the abundance of the established surface markers CD19, CD27, IgD, CD38. The following subpopulations were defined: Total B cells CD19+, memory B cells CD19+CD27+, unswitched memory B cells CD19+CD27+IgD+, switched memory B cells CD19 +CD27+IgD- and plasmablasts CD19+CD27++CD38++. Murine splenocytes were gated via CD19, GL7, Fas (CD95) and CD80, with CD19+GL7-Fas- being defined as non-GC B cells, CD19+GL7+Fas+ as GC B cells and CD19+CD80+ as CD80+ memory B cells. Nuclear Translocation was assessed using the computed “SimilarityDilate” (co-localization score) of the nuclear signal (7-AAD) and the TFEB (or NF-kB p50, p65 or NFAT1) signal. Cells with a "SimilarityDilate" >1.0 were considered as nuclear positive for the respective transcription factor. Gating strategies are depicted within the corresponding (supplementary) figures.
Flow cytometric analysis of intracellular and surface markers was carried out on single cells according to FSC-A/SSC-A, followed by FSC-A/FSC-H assessment. Due to the homogeneity of cultured cell lines, cells were nor further divided into subpopulations. Murine splenocytes were gated for ZombieAqua negativity and for the B cell markers CD19, GL7 and Fas (CD95) with CD19+GL7-Fas- defined as non-GC B cells and CD19+GL7+Fas+ as GC B cells. Gating strategies are depicted within the corresponding (supplementary) figures.

☒ Tick this box to confirm that a figure exemplifying the gating strategy is provided in the Supplementary Information.
